# Biotin Induces Inactive Chromosome X Reactivation and Corrects Physiopathological Alterations in Beta-Propeller-Protein-Associated Neurodegeneration

**DOI:** 10.3390/ijms26031315

**Published:** 2025-02-04

**Authors:** Diana Reche-López, Ana Romero-González, Mónica Álvarez-Córdoba, Alejandra Suárez-Carrillo, Paula Cilleros-Holgado, Rocío Piñero-Pérez, David Gómez-Fernández, José Manuel Romero-Domínguez, Alejandra López-Cabrera, Susana González-Granero, José Manuel García-Verdugo, José A. Sánchez-Alcázar

**Affiliations:** 1 Centro Andaluz de Biología del Desarrollo (CABD-CSIC-Universidad Pablo de Olavide), 41013 Sevilla, Spain; dreclop@alu.upo.es (D.R.-L.); aromgon1@upo.es (A.R.-G.); malvcor@upo.es (M.Á.-C.); asuacar1@alu.upo.es (A.S.-C.); pcilhol@alu.upo.es (P.C.-H.); rpieper@alu.upo.es (R.P.-P.); dgomfer1@acu.upo.es (D.G.-F.); jmromdom@upo.es (J.M.R.-D.); alopcab2@alu.upo.es (A.L.-C.); 2 Laboratory of Comparative Neurobiology, Cavanilles Institute of Biodiversity and Evolutionary Biology, University of Valencia and CIBERNED-ISCIII, 46980 Valencia, Spain; susana.gonzalez@uv.es (S.G.-G.); j.manuel.garcia@uv.es (J.M.G.-V.)

**Keywords:** autophagy, biotin, BPAN, direct reprogramming, epigenetic nutrients, iron accumulation, NBIA, WDR45, Xist, X-linked diseases

## Abstract

Neurodegeneration with brain iron accumulation (NBIA) involves a group of rare neurogenetic disorders often linked with iron overload in the basal nuclei of the brain presenting with spasticity, dystonia, muscle rigidity, neuropsychiatric symptoms, and retinal degeneration. Among NBIA subtypes, beta-propeller-protein-associated neurodegeneration (BPAN) is associated with mutations in the autophagy gene WDR45 (WD repeat domain 45). Previously, we demonstrated that WDR45 mutations in BPAN cellular models impaired autophagy, iron metabolism, and cell bioenergetics. In addition, antioxidant supplementation partially improved cell physiopathology; however, autophagy and cell bioenergetics remained affected. In this work, we explored the possibility of expressing the normal WDR45 allele present in the inactive chromosome X (Xi) of BPAN cells through treatment with epigenetic modulators. The aim of this study was to demonstrate whether biotin, an epigenetic nutrient, was able to restore the expression levels of WDR45 by a mechanism involving Xi reactivation and, consequently, correct BPAN defects. Our study demonstrated that biotin supplementation increases histone biotinylation and allows for the transcription of the WDR45 allele in Xi. Consequently, all physiopathological alterations in BPAN cells were notably corrected. The reactivation of Xi by epigenetic modulators can be a promising approach for the treatment of BPAN and other X-linked diseases.

## 1. Introduction

Iron is an essential trace element crucial for various cellular processes, particularly in the brain, which is the most metabolically active organ in the body. It plays a vital role in oxidative metabolism, myelination, mitochondrial energy production, and neurotransmitter biosynthesis [1,2]. Nevertheless, excess iron can be harmful, generating highly reactive free radicals through the Fenton reaction, leading to oxidative stress that damages DNA, lipids, and proteins. Consequently, maintaining precise iron homeostasis is critical for the brain to prevent the accumulation of toxic iron levels while ensuring adequate supply for its essential functions [3].

One of the most notable disorders involving iron accumulation in the brain is neurodegeneration with brain iron accumulation (NBIA), a diverse group of neurodegenerative diseases. A recently identified subtype of NBIA is β-propeller-protein-associated neurodegeneration (BPAN), also classified as type 5 NBIA. BPAN is a rare condition that combines both neurodevelopmental and neurodegenerative features, marked by brain iron accumulation [4].

BPAN is caused by de novo pathogenic variants in the *WDR45* (WD repeat domain 45) gene, which follows an X-linked dominant inheritance pattern. The *WDR45* gene encodes a beta-propeller protein involved in lysosomal autophagy and endoplasmic reticulum (ER) homeostasis [5,6]. Missense and truncation mutations in *WDR45* result in the loss of protein function [7,8].

WDR45 protein is a member of the WIPI (WD repeat domain, phosphoinositide-interacting) family involved in several key cellular processes. While the precise function of WDR45 remains unclear, its disruption leads to defects in autophagy, mitochondrial dysfunction, ER stress, and abnormal iron homeostasis. All those processes contribute to BPAN pathology [9].

WDR45 is particularly critical for autophagy, a fundamental process for the degradation and recycling of damaged cellular components [10]. Studies have shown that the deficiency of WDR45 in BPAN patients and animal models results in impaired autophagic flux [11,12]. Recent research has indicated that WDR45 deficiency may disrupt iron homeostasis, particularly in fibroblasts from BPAN patients, where iron levels were found to be elevated [13]. Additionally, studies of fibroblasts and neurons derived from a female BPAN patient have revealed mitochondrial dysfunction and lysosomal impairments, suggesting that WDR45 is implicated in the regulation of iron metabolism [14]. However, the mechanism by which WDR45 deficiency leads to iron accumulation in the brain is still largely unknown.

Children with BPAN often present developmental delays, seizures, and sleep disturbances [7,15,16], and, later, they commonly develop dementia and parkinsonism [17]. Though male cases are rare, the disorder was once thought to be embryonically lethal in males [8,18]. In females, the disorder can still manifest due to X-chromosome inactivation, where only one of the two X-chromosomes is expressed in each cell. The advent of next-generation sequencing has recently revealed a broader spectrum with less severe outcomes, suggesting that BPAN may span a continuum of phenotypic manifestations with varied clinical results [7]. Diagnosing BPAN remains challenging and typically requires whole-exome sequencing to confirm the genetic mutation [19].

In *wdr45*-knockout (KO) cells, the loss of WDR45 results in defects in ferritinophagy, a selective autophagic degradation of ferritin, leading to iron accumulation, impaired mitochondrial respiration, increased reactive oxygen species (ROS), and enhanced cell death. Iron accumulation was also detected in the mitochondria [20].

Mouse models have additionally demonstrated the importance of WDR45 in neuronal development and survival. In *wdr45*-KO mice, defects in motor coordination, learning, and memory, as well as axon swelling, have been observed [12]. Mice neurons exhibit autophagy defects, with the accumulation of ubiquitin-positive aggregates in both neurons and axons. Furthermore, *wdr45*-KO mice display cognitive impairments, abnormal synaptic transmission, and lesions in several brain regions, with an associated increase in ER stress and neuronal apoptosis [14], which aligns with the neurodegeneration observed in BPAN patients.

Our previous work demonstrated reduced WDR45 protein and mRNA levels in fibroblasts derived from BPAN patients. Additionally, treatments with inducers or inhibitors of autophagy do not increase microtubule-associated protein 1 light chain 3 beta (LC3B), a protein involved in the selection of autophagy substrates and autophagosome biogenesis in mutant cells, indicating impaired autophagic flux due to decreased autophagosome formation. Mitochondrial vacuolization and accumulation of lipofuscin-like aggregates was observed via transmission electron microscopy (TEM). BPAN cells also exhibited altered mitochondrial bioenergetics, lipid peroxidation, and iron accumulation. Antioxidant treatments (vitamin E, pantothenate and alpha-lipoic acid) were found to prevent lipid peroxidation and iron accumulation, though they did not restore WDR45 expression or correct the autophagic defect or mitochondrial bioenergetics [21].

From the therapeutic point of view, one of the objectives would be to increase the expression levels of *WDR45* by increasing the transcription and protein stability of mutant *WDR45* or enhancing the transcription of the *WDR45* in the inactive chromosome X (Xi) in females by a mechanism known as chromosome Xi reactivation [22,23]. A cure may be found within the patient’s own cells because every cell carries a wild-type copy of the affected gene on Xi.

Therefore, the selective reactivation of Xi could be a viable approach to address the root cause of BPAN and other X-linked disorders such as Rett syndrome, CDKL5 deficiency disorder, Fragile X syndrome, and many other X-linked intellectual disabilities [24]. Among these disorders, Rett syndrome has recently attracted significant attention for the use of epigenetic mechanisms linked to Xi reactivation in its treatment [22]. Biotin, a nutritional epigenetic regulator also known as vitamin B7 or vitamin H, is a water-soluble B-complex vitamin with a unique bicyclic structure. Recently, biotin has emerged as a significant epigenetic regulator due to the ability of biotin to covalently bind to lysine residues in histones. This modification, termed histone biotinylation, highlights its role in chromatin remodeling and gene expression regulation [25,26]. Consequently, this study aimed to evaluate the therapeutic effect of biotin on pathological alterations and protein expression levels of wild-type WDR45 by inducing selective Xi reactivation in BPAN cellular models.

## 2. Results

### 2.1. Biotin Supplementation Increases WDR45 Protein and Transcript Expression Levels in BPAN Fibroblasts

**Figure 1:**
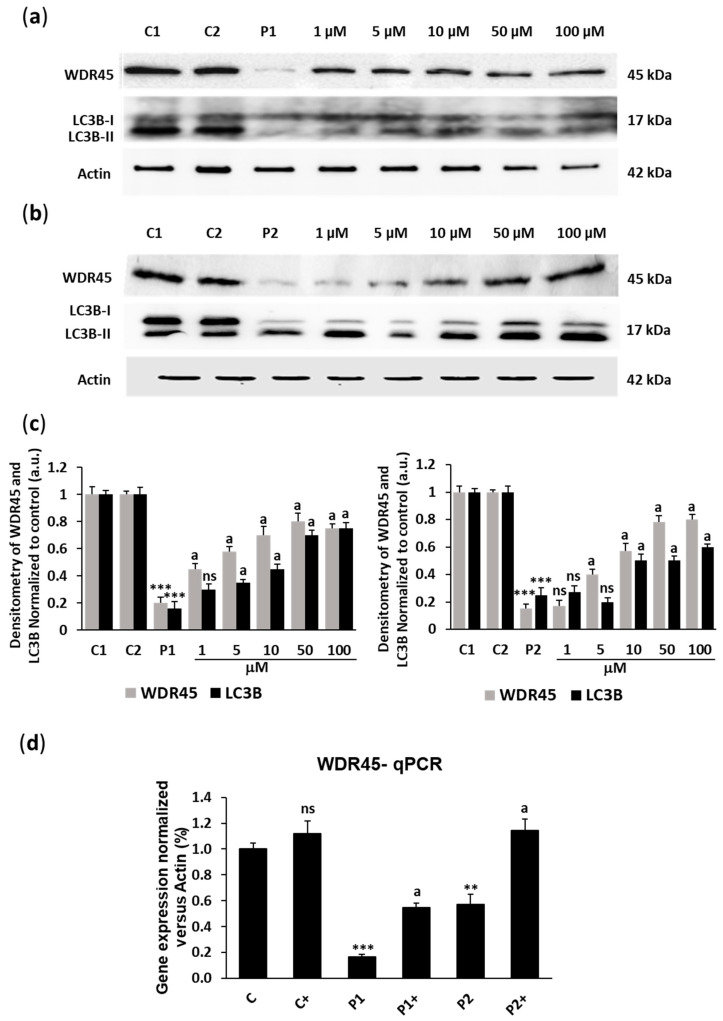
Dose effect of biotin supplementation on WDR45 and LC3B expression levels. (**a**,**b**) Immunoblotting analysis of cellular extracts from control (C1 and C2) and BPAN patient (P1 and P2) cell lines untreated and treated (+) with 1, 5, 10, 50, and 100 μM biotin for one week. Protein extracts (20 μg) were separated on an SDS polyacrylamide gel and immunostained with antibodies against WDR45 and LC3B. Actin was used as a loading control. (**c**) Densitometry of the Western blotting. (**d**) *WDR45* gene expression in control (C) and BPAN (P1 and P2) cells untreated and treated (+) with 10 μM biotin for one week, quantified by RT-qPCR. Results were normalized to actin and referred to control. Data represent the mean ± SD of three separate experiments. ** *p* < 0.01, and *** *p* < 0.001 between BPAN and control cells. ^a^ *p* < 0.01 between the presence and the absence of biotin; a.u.: arbitrary units; ns: not significant.

First, we analyzed the dose effect of biotin supplementation on WDR45 expression levels in fibroblast cell lines derived from two BPAN patients. As shown by Western blot analysis in Figure 1a,b, WDR45 expression levels (band around 45 kDa) were markedly reduced in patients P1 and P2 and significantly increased in a dose-dependent manner after biotin treatment (Figure 1a,b). As the P1 patient harbors a stop codon mutation in the mutant allele, the increased levels of WDR45 suggested that biotin supplementation in P1 was inducing the expression of the normal allele at the inactive chromosome X. Interestingly, increased levels of WDR45 were associated with a marked increase in the lipid conjugated form of the protein LC3 (LC3-II) (Figure 1a,b), an essential autophagosome marker for its formation, elongation, and closure, suggesting the restoration of autophagosome formation in BPAN cells. As a biotin concentration at 10 µM was the minimum positive concentration in both cell lines, we chose this concentration for the next experiments in this study.

The low protein expression levels of WDR45 in BPAN cells were also associated with a reduction in *WDR45* transcript levels, suggesting a decrease in *WDR45* gene expression (Figure 1d). Biotin supplementation (10 µM) significantly increased *WDR45* gene expression in both P1 and P2 fibroblasts.

### 2.2. Biotin Supplementation Induces Inactive Chromosome X Reactivation and Increases WDR45 Transcript Expression in BPAN Fibroblasts

**Figure 2:**
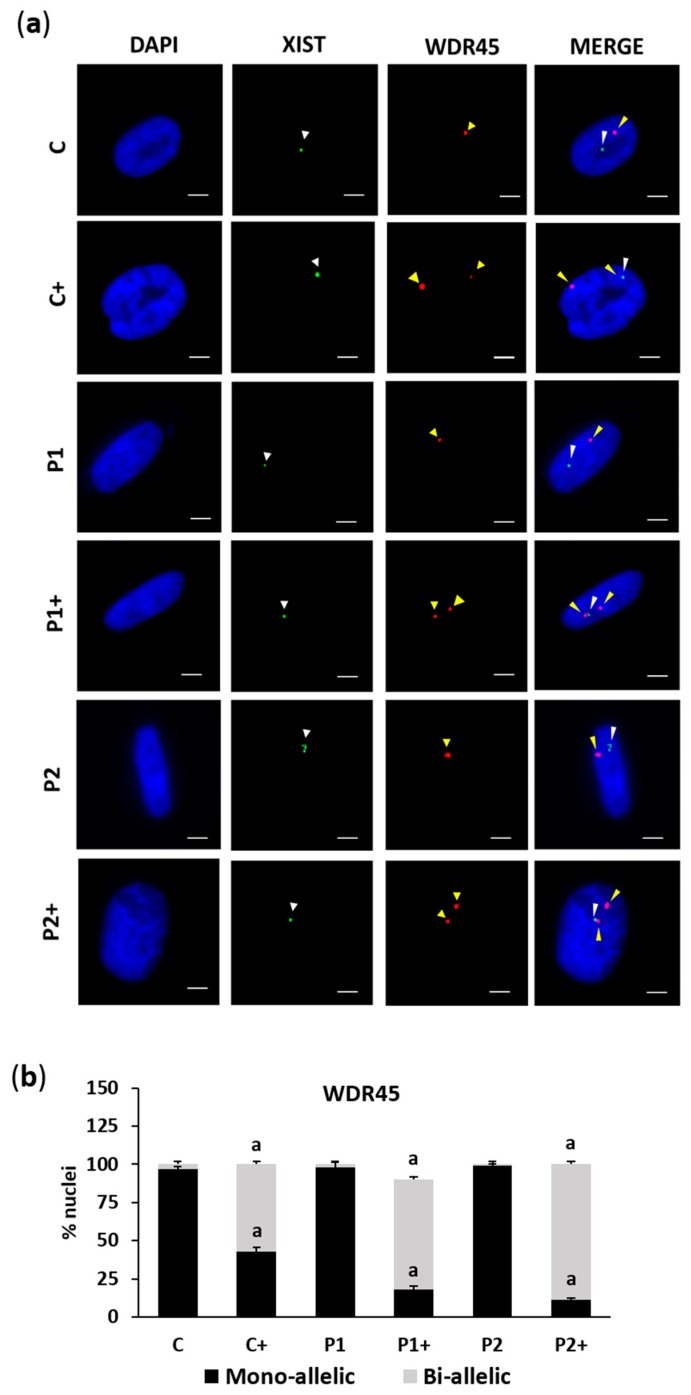
Effect of biotin supplementation on Xi reactivation. (**a**) Representative images of two-color RNA FISH of control (C) and BPAN (P1 and P2) fibroblasts untreated and treated (+) with 10 μM biotin for one week, monitoring the expression of *Xist* (green) and *WDR45* (red). Nuclei were stained with 1 μg/mL DAPI (blue). The white arrowheads indicate *Xist* expression, and the yellow arrowheads indicate *WDR45* signals. Scale bar: 20 µm. (**b**) Percentage of monoallelic and biallelic *WDR45* expression; 100 nuclei were examined per condition and only cells with RNA FISH signal were analyzed. Data represent the mean ± SD of three separate experiments. ^a^ *p* < 0.01 between the presence and the absence of biotin.

To address the effect of biotin on Xi reactivation in BPAN fibroblasts, we analyzed the expression of *WDR45* and *X-inactive-specific transcript* (*Xist*), a marker of Xi, using two-color RNA fluorescence in situ hybridization (FISH). RNA FISH revealed, as expected, a single nuclear signal for *WDR45* and *Xist* in control (C) and BPAN (P1 and P2) fibroblasts, indicative of monoallelic expression of *WDR45* (Figure 2a,b). Biotin supplementation (10 µM) substantially increased the fraction of cells containing two nuclear *WDR45* signals, indicative of biallelic expression. Interestingly, one of the *WDR45* signals was near the *Xist* signal, suggesting Xi reactivation.

### 2.3. Biotin Supplementation Increases Histone Biotinylation in BPAN Fibroblasts

**Figure 3:**
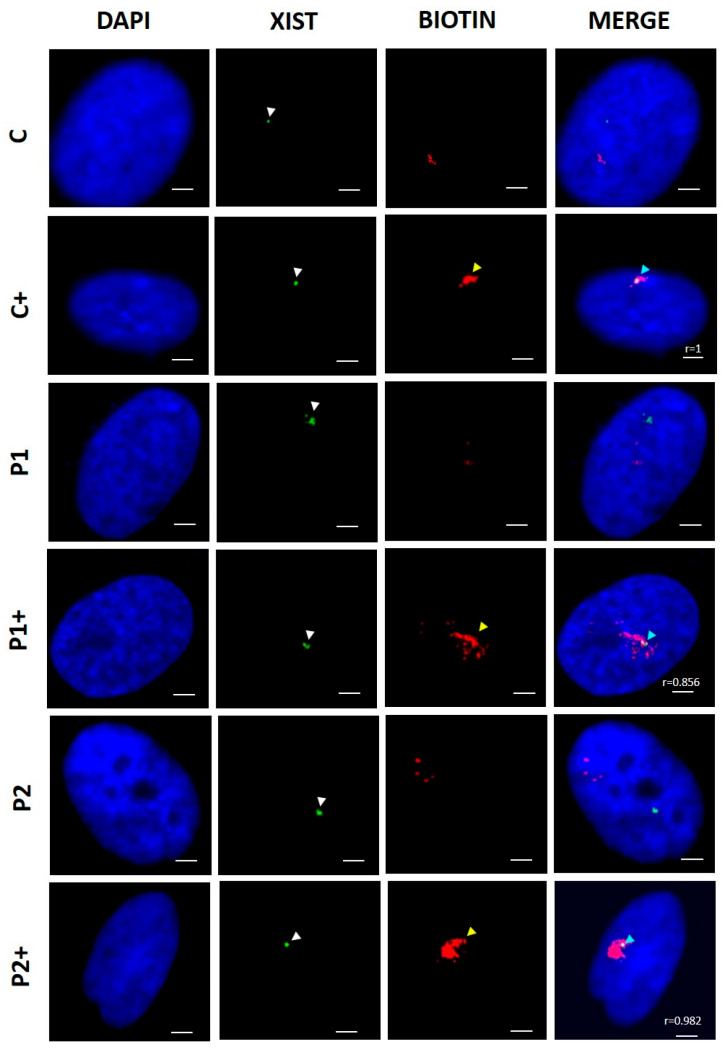
Effect of biotin supplementation on nuclei biotinylation. Representative images of two-color RNA FISH of control (C) and BPAN (P1 and P2) fibroblasts untreated and treated (+) with 10 μM biotin for one week, monitoring the expression of *Xist* (green) and anti-biotin antibody signal (red). Nuclei were stained with 1 μg/mL DAPI (blue). The white arrowheads indicate *Xist* expression, the yellow arrowheads indicate anti-biotin signals, and the light-blue arrowheads indicate colocalization of *Xist* and biotin signals. Scale bar: 20 µm. To corroborate *Xist* presence in the anti-biotin antibody, we calculated the Pearson correlation coefficient. Positive correlation was considered with a Pearson coefficient > 0.75; 100 nuclei were examined per condition and only cells with RNA FISH signal were analyzed. Data represent the mean ± SD of three separate experiments.

As, theoretically, the biotinylation of histones might lead to chromatin remodeling and the increased transcription of DNA in analogy with the transcriptional activation of DNA via the acetylation and ubiquitination of histones [27,28,29], we next examined whether biotin supplementation induced chromosome Xi reactivation by increasing the histone biotinylation of DNA regions of the condensed chromosome. To evaluate this hypothesis, we performed an RNA FISH assay to detect Xi by detecting *Xist* signal in combination with an immunofluorescence staining of biotin for detecting the distribution patterns of biotinylated histones (Figure 3). The results showed that the *Xist* signal colocalized with areas of biotin signal in control (C) and BPAN (P1 and P2) fibroblasts supplemented with biotin (10 µM), suggesting that Xi reactivation by biotin supplementation may be due to the histone biotinylation of the condensed chromosome.

**Figure 4:**
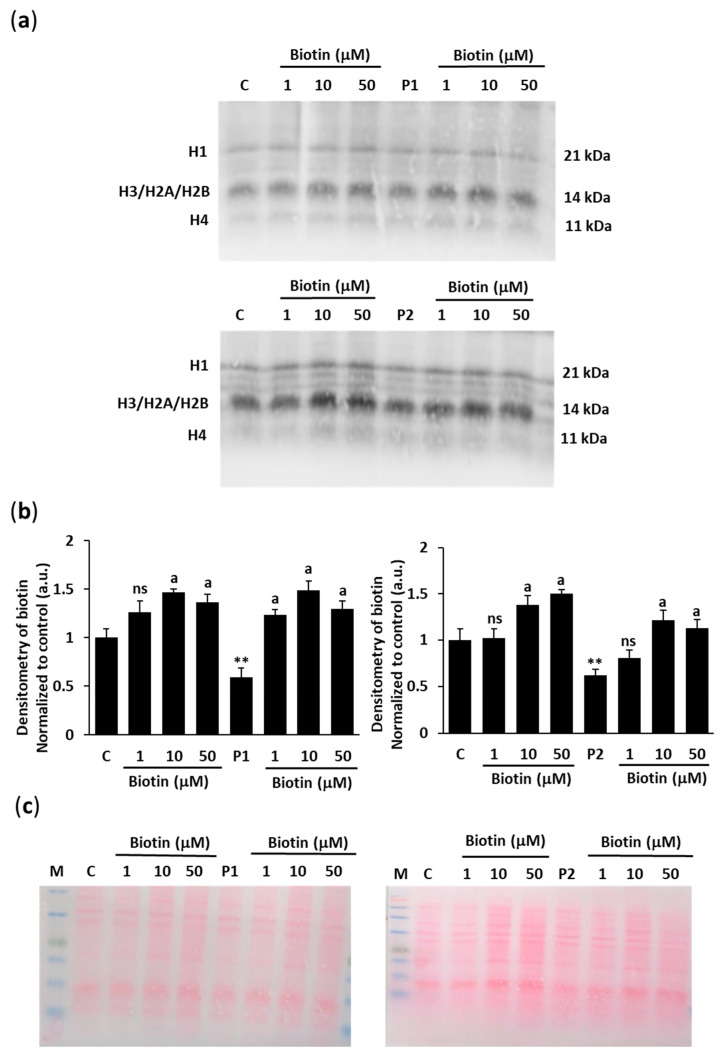
Effect of biotin supplementation on the biotinylation of histones in cell nuclei. (**a**) Immunoblotting of biotin protein expression levels in histones H1, H2A, H3, and H4 of control (C) and BPAN (P1 and P2) cell lines untreated and treated (+) with 1, 10, and 50 µM biotin for one week. Histones were isolated using histone extraction kit. Nuclei extracts (10 μg) were separated on an SDS polyacrylamide gel and immunostained with a monoclonal antibody against biotin. (**b**) Densitometry of Western blotting. (**c**) Ponceau S staining as loading control. Results were referred to control. Data represent the mean ± SD of three separate experiments. ** *p* < 0.01 between BPAN and control cells. ^a^ *p* < 0.01 between the presence and the absence of biotin; a.u.: arbitrary units; ns: not significant.

To confirm histone biotinylation under increased concentrations of biotin supplementation, histones were isolated from nuclei of control (C) and BPAN (P1 and P2) cells by histone extraction kit and biotinylated histones were then detected by Western blotting. We used polyacrylamide gels to identify the five major classes of histones (H1, H2A, H2B, H3, and H4). Extracts from cell nuclei were then used to determine whether histones contained covalently bound biotin using a monoclonal antibody against biotin (Figure 4a,b). The results showed that the abundance of biotinylated histones H1, H2A, H2B, H3, and H4 in the cell nuclei increased significantly with biotin concentrations of 1, 10, and 50 µM in the medium after 1 week of culture. The absence of quantitatively important nonhistone bands after Ponceau staining further confirmed the purity of the preparation (Figure 4c).

**Figure 5:**
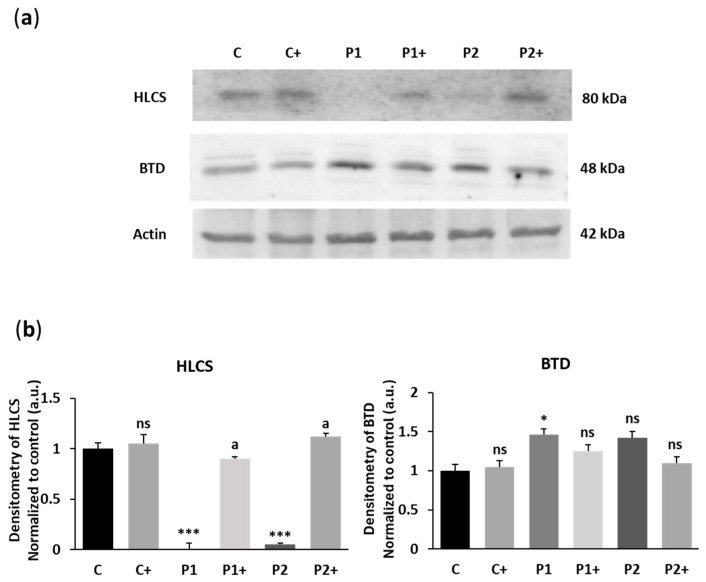
T Effect of biotin supplementation on HLCS and BTD expression levels. (**a**) Immunoblotting of HLCS and BTD protein expression levels of control (C) and BPAN (P1 and P2) cell lines untreated and treated (+) with 10 µM biotin for one week. Protein extracts (20 μg) were separated on an SDS polyacrylamide gel and immunostained with antibodies against HLCS and BTD. Actin was used as a loading control. (**b**) Densitometry of the Western blotting. Results were normalized to actin and referred to control. Data represent the mean ± SD of three separate experiments. * *p* < 0.05, and *** *p* < 0.001 between BPAN and control cells. ^a^ *p* < 0.01 between the presence and the absence of biotin; a.u.: arbitrary units; ns: not significant.

Furthermore, as the histone biotinylation pattern is mediated by the activity of biotinidase (BTD) and holocarboxylase synthetase (HLCS) [30], the expression levels of both enzymes were also examined by Western blotting under biotin supplementation (Figure 5a,b). Biotin supplementation induced a significant increase in HLCS expression levels, consistent with the up-regulation of the enzyme directly involved in protein biotinylation. However, no significant changes in BTD expression levels were observed.

### 2.4. Biotin Treatment Restores Autophagosome Formation and Lysosome Acidification in BPAN Fibroblasts

**Figure 6:**
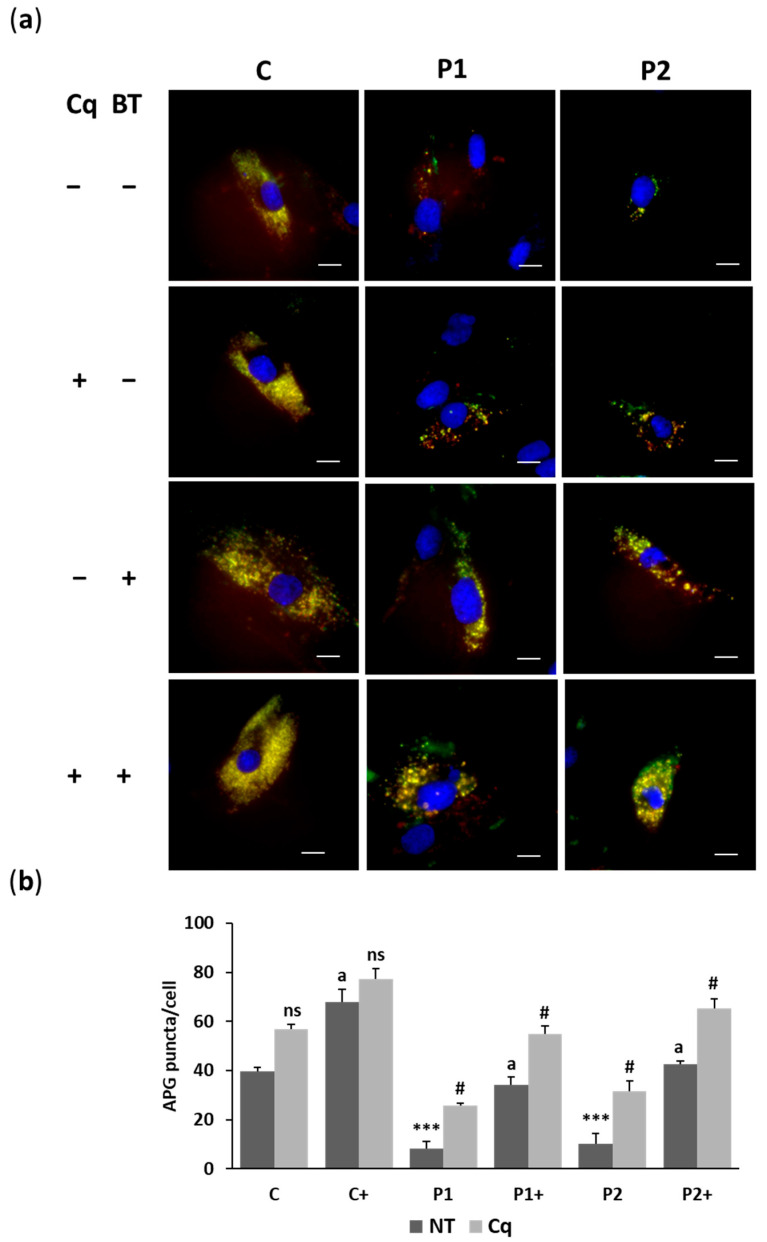
Effect of biotin supplementation on autophagosome formation in basal conditions and after autophagy inhibition by Cq. (**a**) Representative fluorescence microscopy images of neutral pH autophagosomes (yellow) of control (C) and BPAN (P1 and P2) fibroblasts untreated and treated (+) with 10 μM biotin (BT) for one week and incubated with 90 μM Cq for 16 h. Nuclei were stained with 1 μg/mL DAPI (blue). Scale bar: 20 µm. (**b**) Quantification of neutral pH autophagosomes (RFP + GFP positive puncta). At least 100 cells per condition were analyzed. Data represent the mean ± SD of three separate experiments. *** *p* < 0.001 between BPAN and control cells. ^a^ *p* < 0.01 between the presence and the absence of biotin. ^#^ *p* < 0.01 between the presence and the absence of Cq. APG: autophagosomes; ns: not significant; NT: not treated.

**Figure 7:**
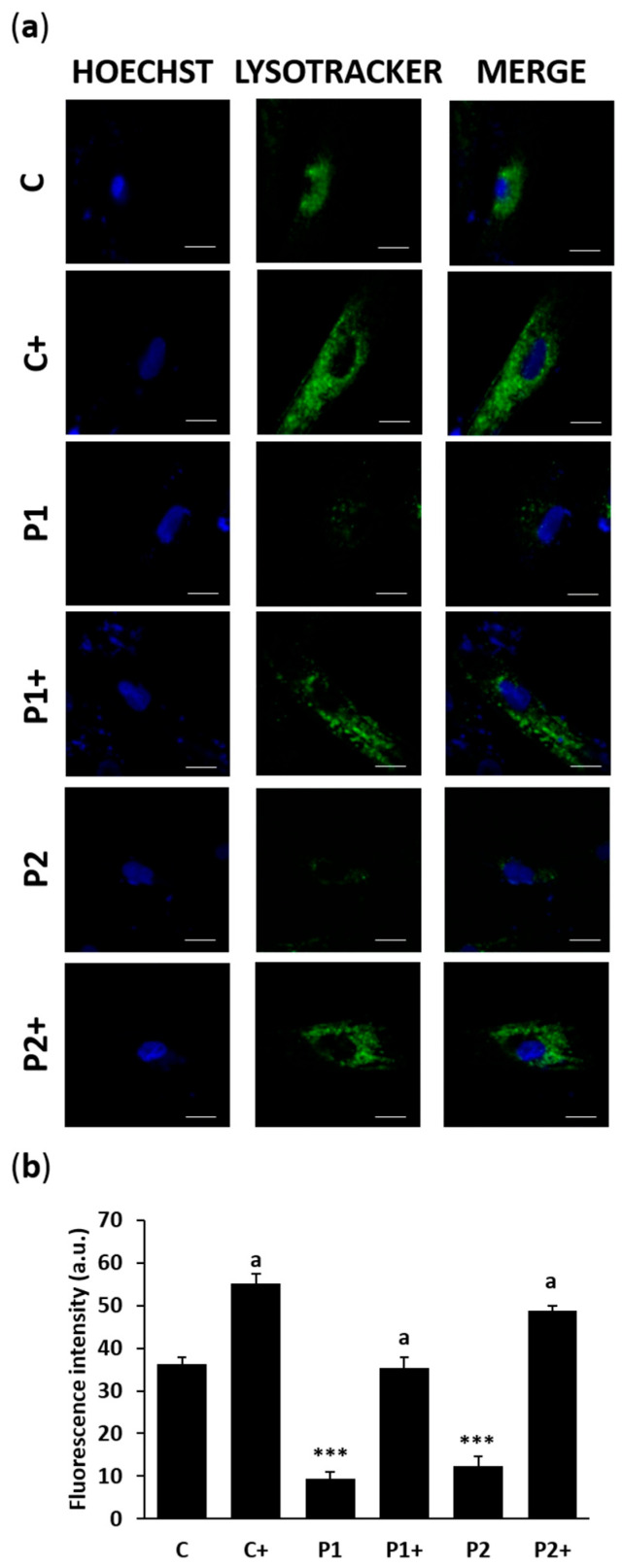
Effect of biotin supplementation on lysosomal compartment. (**a**) Representative fluorescence images of Lysotracker staining (green) of control (C) and BPAN (P1 and P2) fibroblasts untreated and treated (+) with 10 μM biotin for one week and incubated with 75 nM Lysotracker for an hour. Nuclei were revealed by Hoechst 33,342 staining (blue). Scale bar: 20 µm. (**b**) Lysotracker fluorescence intensity quantification. At least 100 cells per condition were analyzed. Data represent the mean ± SD of three separate experiments. *** *p* < 0.001 between BPAN and control cells. ^a^ *p* < 0.01 between the presence and the absence of biotin; a.u.: arbitrary units.

To evaluate the impact of biotin on autophagy defects, we employed the Tandem Sensor RFP-GFP-LC3B for monitoring autophagosomes and autolysosomes formation in untreated and treated control and BPAN fibroblasts (Figure 6a,b and Figure S1). The results demonstrated that biotin (10 µM) significantly enhanced autophagosome formation, even in the presence of chloroquine (Cq), which induces the accumulation of autophagosomes. This suggests that biotin may improve the autophagic dysfunction observed in BPAN cells. Additionally, lysotracker staining showed that biotin supplementation notably restored the lysosomal content/acidification in BPAN fibroblasts (Figure 7a,b).

### 2.5. Biotin Supplementation Improves Mitophagy Activity in BPAN Fibroblasts

**Figure 8:**
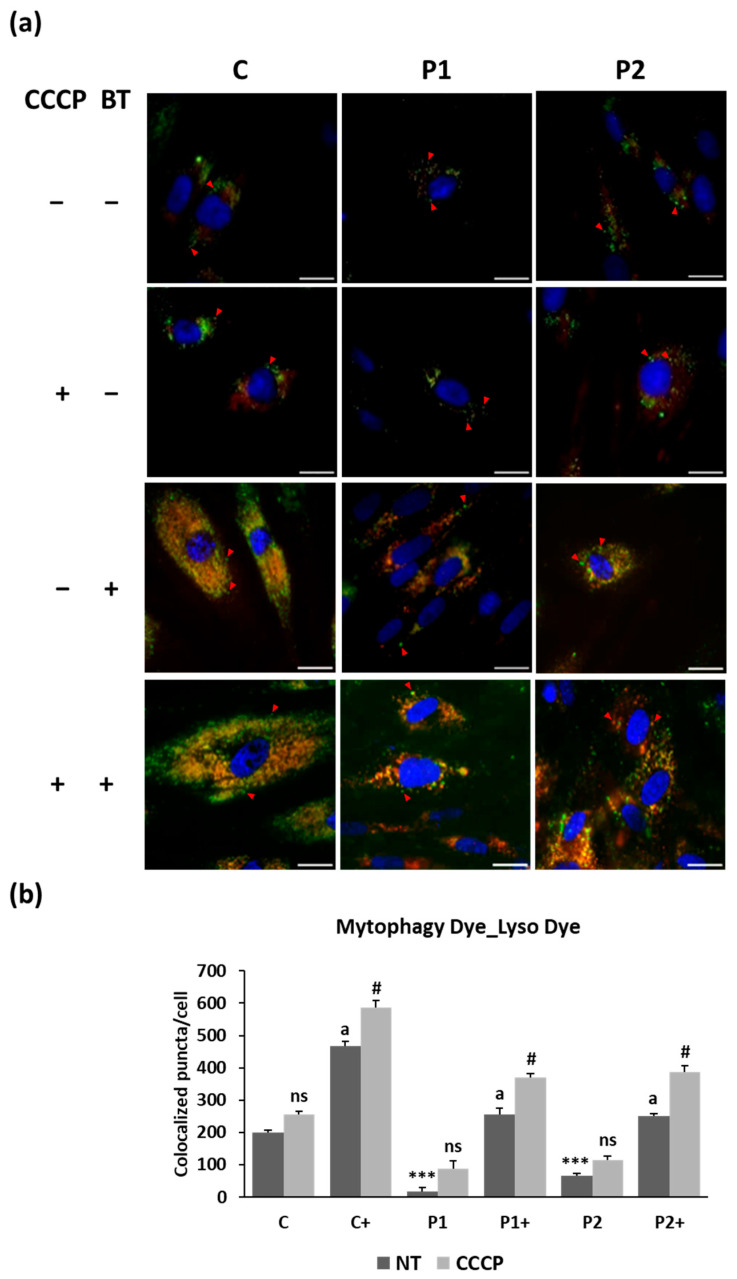
The effect of biotin supplementation on mitophagy activity. (**a**) Representative fluorescence images of puncta (red arrows) Mitophagy Dye and Lyso Dye merge (yellow) of control (C) and BPAN (P1 and P2) fibroblasts untreated and treated (+) with 10 μM biotin for one week and incubated with 10 µM CCCP for 4 h. Nuclei were revealed by Hoechst 33,342 staining (blue). Scale bar: 20 µm. (**b**) Mitophagy Dye/Lyso Dye puncta/cell quantification. At least 100 cells per condition were analyzed. Data represent the mean ± SD of three separate experiments. *** *p* < 0.001 between BPAN and control cells. ^a^ *p* < 0.01 between the presence and the absence of biotin. ^#^ *p* < 0.01 between the presence and the absence of CCCP; ns: not significant; NT: not treated.

Given that BPAN fibroblasts exhibit deficient autophagy, the mitophagy pathway seems to be disrupted, resulting in compromised mitochondrial renewal and function [31]. To corroborate mitophagy failure and the effect of biotin supplementation on BPAN cells, we performed a fluorescence assay using a mitophagy detection kit (Figure 8a,b and Figure S2). This kit is composed of Mitophagy Dye, for mitophagy detection, and Lyso Dye, for lysosomal detection. Mitophagy Dye localizes to intact mitochondria and exhibits a weak fluorescence, but emits a high fluorescence when mitophagy is induced and the damaged mitochondria fuses to lysosome. To confirm the fusion of Mitophagy-Dye-labeled mitochondria and lysosome (Lyso Dye), we quantified the colocalization puncta. BPAN cells showed impaired mitophagy even after treatment with the mitochondrial uncoupler carbonyl cyanide m-chlorophenylhydrazone (CCCP), a potent inducer of mitophagy. Interestingly, biotin supplementation significantly increased the number of Mitophagy Dye–Lyso Dye puncta, indicating an improvement in autophagy/mitophagy activity.

### 2.6. Biotin Treatment Increases Mitochondrial Bioenergetics in BPAN Fibroblasts

**Figure 9:**
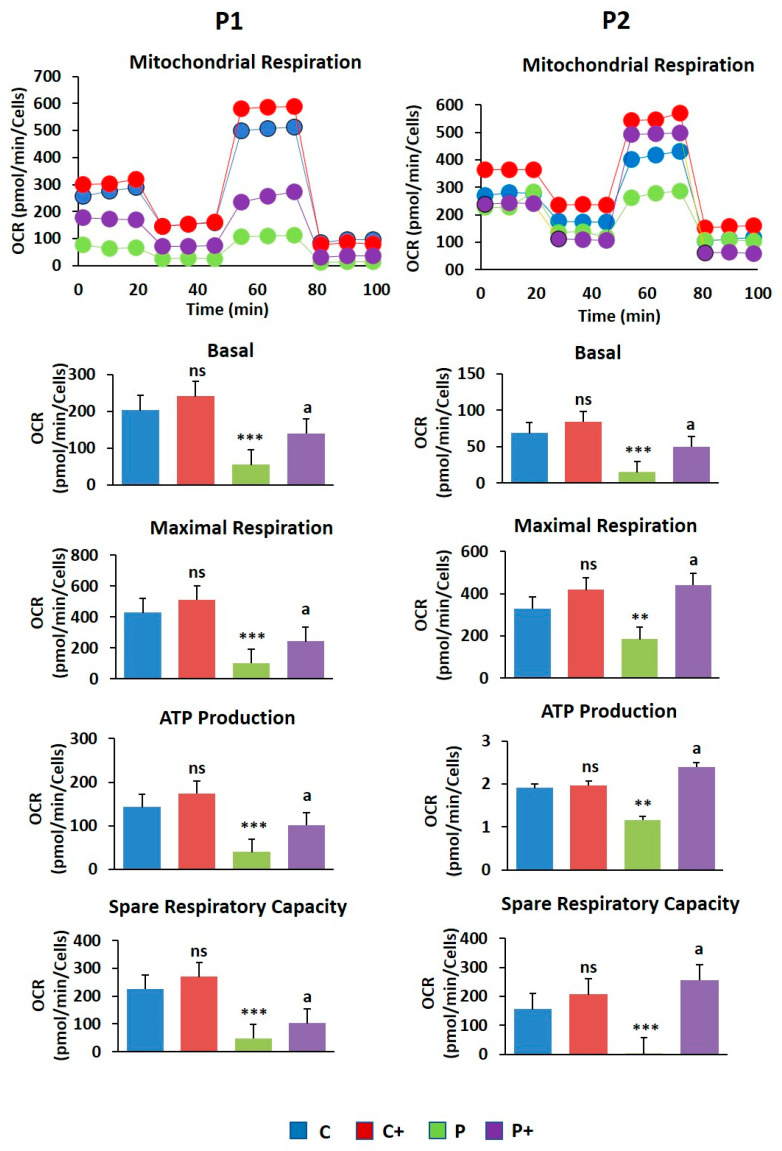
Effect of biotin on cell bioenergetics. Basal and maximal respiration, ATP production, and spare respiratory capacity of control (C) and BPAN (P1 and P2) fibroblasts untreated and treated (+) with 10 µM biotin for one week using the Seahorse analyzer, as described in Material and Methods. Data represent the mean ± SD of three separate experiments. ** *p* < 0.005, and *** *p* < 0.0005 between BPAN and control cells. ^a^ *p* < 0.01 between the presence and the absence of biotin; ns: not significant.

The oxygen consumption rate (OCR) was measured in treated and untreated control and BPAN fibroblasts using the bioenergetic profile from the Seahorse analyzer to evaluate the biotin effect on mitochondrial function (Figure 9). Biotin supplementation (10 µM) significantly improved the reduced basal, maximal, and spare respiration as well as mitochondrial ATP production in BPAN fibroblasts.

**Figure 10:**
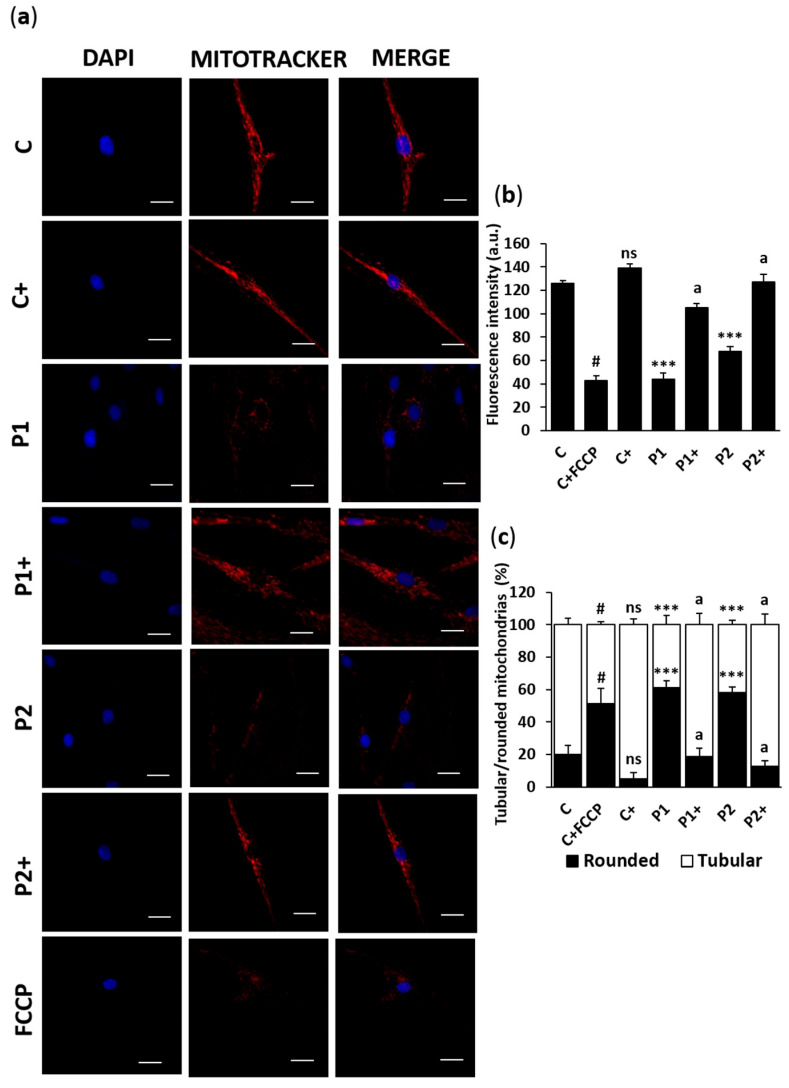
Effect of biotin treatment on mitochondrial polarization and mitochondrial network. (**a**) Representative fluorescence images of mitochondrial polarization and mitochondrial network of control (C) and BPAN (P1 and P2) fibroblasts untreated and treated (+) with 10 μM biotin for one week and stained for 45 min with 100 nM MitoTracker^TM^ Red CMXRos (red). Nuclei were stained with 1 μg/mL DAPI (blue). Control cells were also incubated with 10 µM FCCP, a mitochondrial uncoupler, for 20 min as a negative control. Scale bar: 20 µm. (**b**) Quantification of MitoTracker^TM^ Red CMXRos fluorescence intensity. (**c**) Percentage of tubular and rounded mitochondria. Rounded mitochondria were defined as 0.2–0.5 µm^2^ and tubular mitochondria as >0.5 µm^2^. At least 100 cells per condition were analyzed. Data represent the mean ± SD of three separate experiments. *** *p* < 0.001 between BPAN and control cells. ^a^ *p* < 0.01 between the presence and the absence of biotin. ^#^ *p* < 0.01 between the presence and the absence of FCCP; a.u.: arbitrary units; ns: not significant.

We also tested the effect of biotin on mitochondrial network morphology using Mitotracker^TM^ Red CMXRos staining (Figure 10a–c). Biotin treatment restored the fragmented mitochondrial network and mitochondrial depolarization in BPAN fibroblasts for P1 and P2.

**Figure 11:**
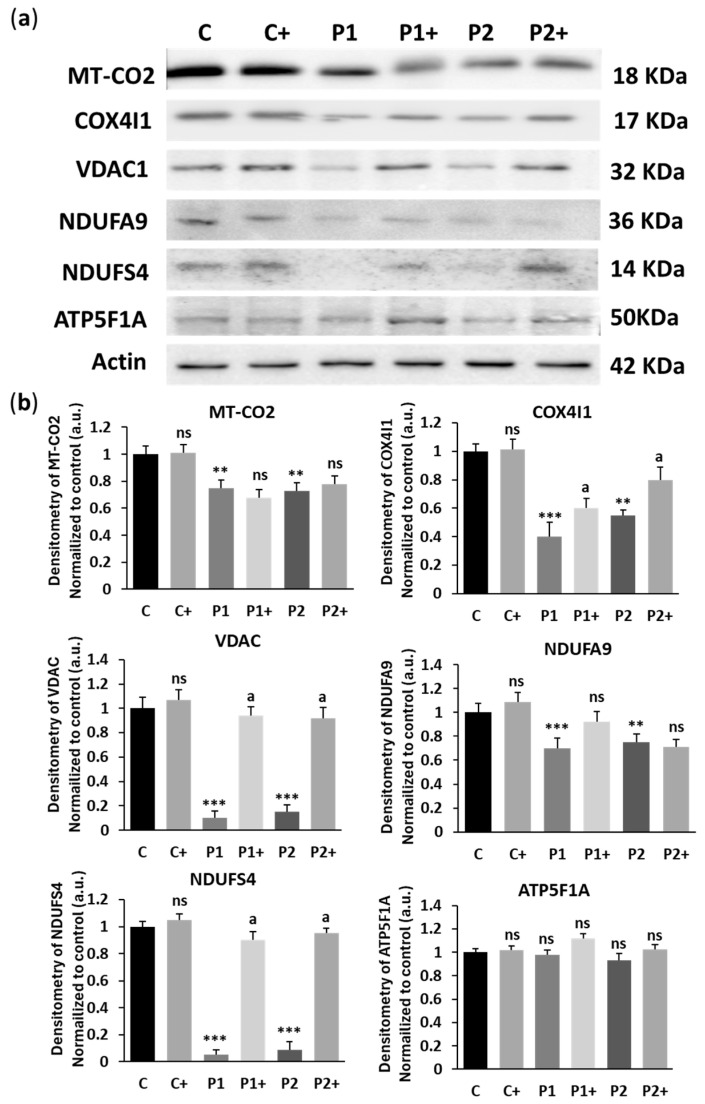
Effect of biotin supplementation on mitochondrial proteins expression levels. (**a**) Immunoblotting of mitochondrial protein expression levels of control (C) and BPAN (P1 and P2) cell lines untreated and treated (+) with 10 μM biotin for one week. Protein extracts (20 μg) were separated on an SDS polyacrylamide gel and immunostained with antibodies against MT-CO2, COX4I1, NDUFS4, NDUFA9, VDAC1, and ATP5F1A. Actin was used as a loading control. (**b**) Densitometry of the Western blotting. Results were normalized to actin and referred to control. Data represent the mean ± SD of three separate experiments. ** *p* < 0.005, and *** *p* < 0.0005 between BPAN and control cells. ^a^ *p* < 0.01 between the presence and the absence of biotin; a.u.: arbitrary units; ns: not significant.

As biotin supplementation increased WDR45 expression levels (Figure 1) and improved autophagy dysfunction (Figure 8), mitochondrial bioenergetics, and morphology (Figure 9 and Figure 10), we next examined its effect on the expression levels of some mitochondrial proteins such as mitochondrially encoded cytochrome c oxidase II (MT-CO2), cytochrome c oxidase subunit 4I1 (COX4I1), NADH:ubiquinone oxidore-ductase subunit A9 (NDUFA9), NADH:ubiquinone oxidoreductase subunit S4 (NDUFS4), voltage-dependent anion channel 1 (VDAC1), and ATP-synthase F1 subunit 1 alpha (ATP5F1A). The results showed that the expression levels of COX4I1, VDAC, and NDUFS4 were significantly increased after biotin supplementation in BPAN cells (Figure 11a,b).

### 2.7. Biotin Supplementation Increases BPAN Fibroblast Viability in Serum-Free Medium

**Figure 12:**
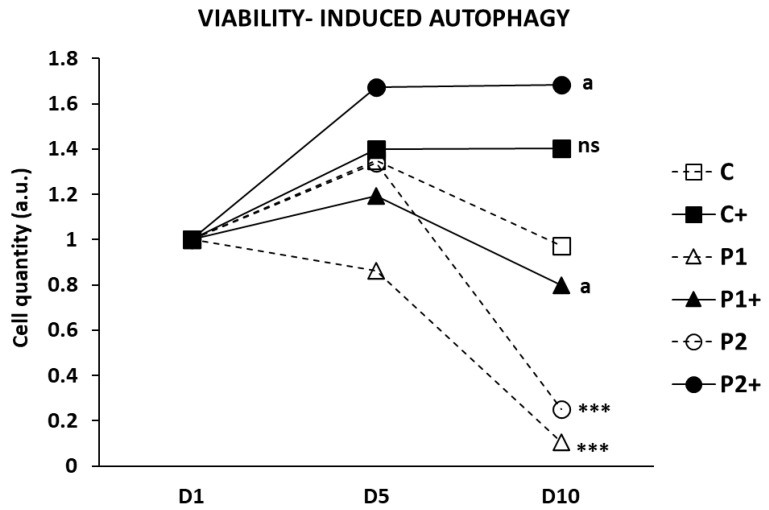
Effect of biotin supplementation on cell viability in serum-free medium. Control (C) and BPAN (P1 and P2) cells untreated and treated (+) with 10 μM biotin were cultured in serum-free medium for 10 days. Cell viability was determined at day 0 (D1), day 5 (D5), and day 10 (D10) by cell counting and trypan blue exclusion method, as described in Material and Methods. Data represent the mean ± SD of three separate experiments. *** *p* < 0.0005 between BPAN cells and controls. ^a^ *p* < 0.01 between the presence and the absence of biotin; a.u.: arbitrary units; ns: not significant.

Under serum starvation conditions, cells initiate autophagy as a survival mechanism [32]. Since BPAN cells exhibit impaired autophagy, reduced viability was observed in the serum-free medium. To investigate if biotin treatment restores autophagy in BPAN cells, we assessed cell viability in control and BPAN fibroblasts with biotin supplementation under these stressful conditions (Figure 12). As anticipated, biotin supplementation significantly improved cell viability during 10 days of serum starvation. These results suggest that, after biotin supplementation, autophagy is functional and plays a protective role in BPAN cells.

### 2.8. Biotin Supplementation Prevents Iron and Lipofuscin Accumulation in BPAN Fibroblasts

**Figure 13:**
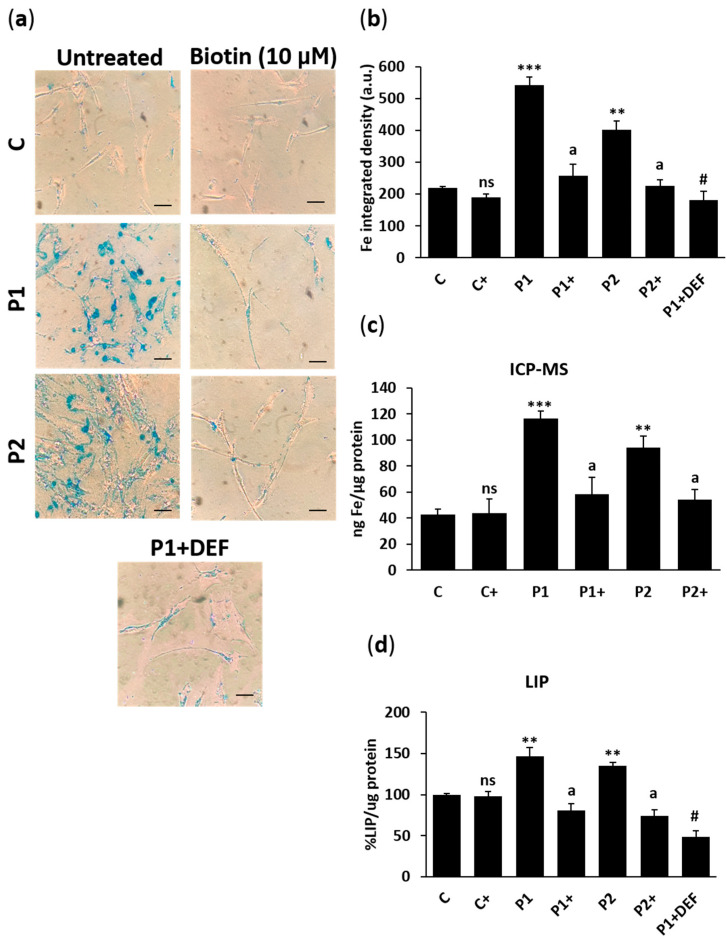
Effect of biotin supplementation on iron metabolism. (**a**) Representative images of Prussian Blue staining of control (C) and BPAN (P1 and P2) fibroblasts untreated and treated (+) with 10 μM biotin for one week. P1 cells were also treated with 100 µM deferiprone (DEF) for 24 h as negative control. Scale bar: 20 μm. (**b**) Quantification of Prussian Blue staining. (**c**) Total iron content determined by ICP-MS. (**d**) Labile iron pool. P1 cells were also treated with 100 µM deferiprone (DEF) for 24 h as negative control. At least 100 cells per condition were analyzed. Data represent the mean ± SD of three separate experiments. ** *p* < 0.01, and *** *p* < 0.001 between BPAN and controls cells. ^a^ *p* < 0.01 between the presence and the absence of biotin. ^#^ *p* < 0.01 between the presence and the absence of deferiprone; a.u.: arbitrary units; ns: not significant.

**Figure 14:**
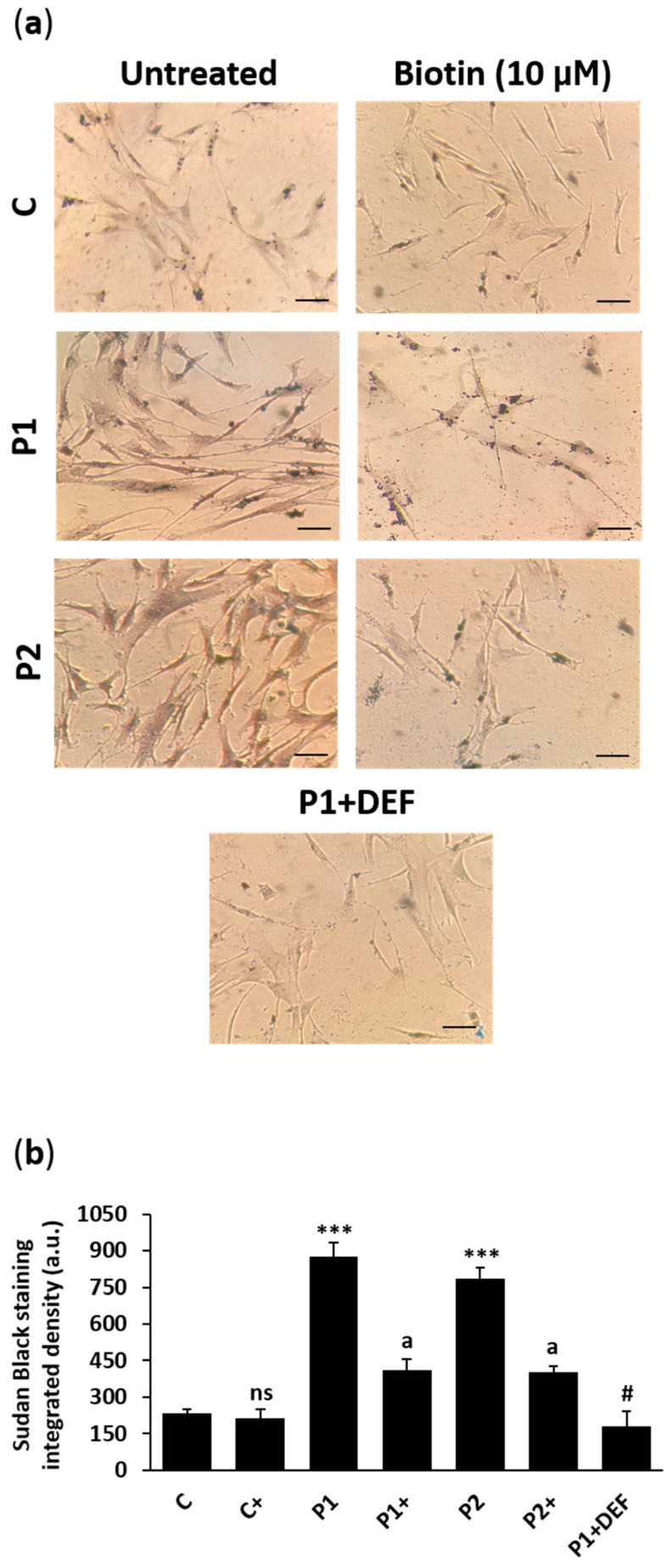
Effect of biotin on lipofuscin accumulation assessed by Sudan Black staining. (**a**) Representative images of Sudan Black staining of control (C) and BPAN (P1 and P2) fibroblasts untreated and treated (+) with 10 μM biotin for one week. P1 cells were also treated with 100 µM deferiprone (DEF) for 24 h as negative control. Scale bar: 20 μm. (**b**) Quantification of Sudan Black staining. At least 100 cells per condition were analyzed. Data represent the mean ± SD of three separate experiments. *** *p* < 0.001 between BPAN and controls cells. ^a^ *p* < 0.01 between the presence and the absence of biotin. ^#^ *p* < 0.01 between the presence and the absence of deferiprone; a.u.: arbitrary units; ns: not significant.

**Figure 15:**
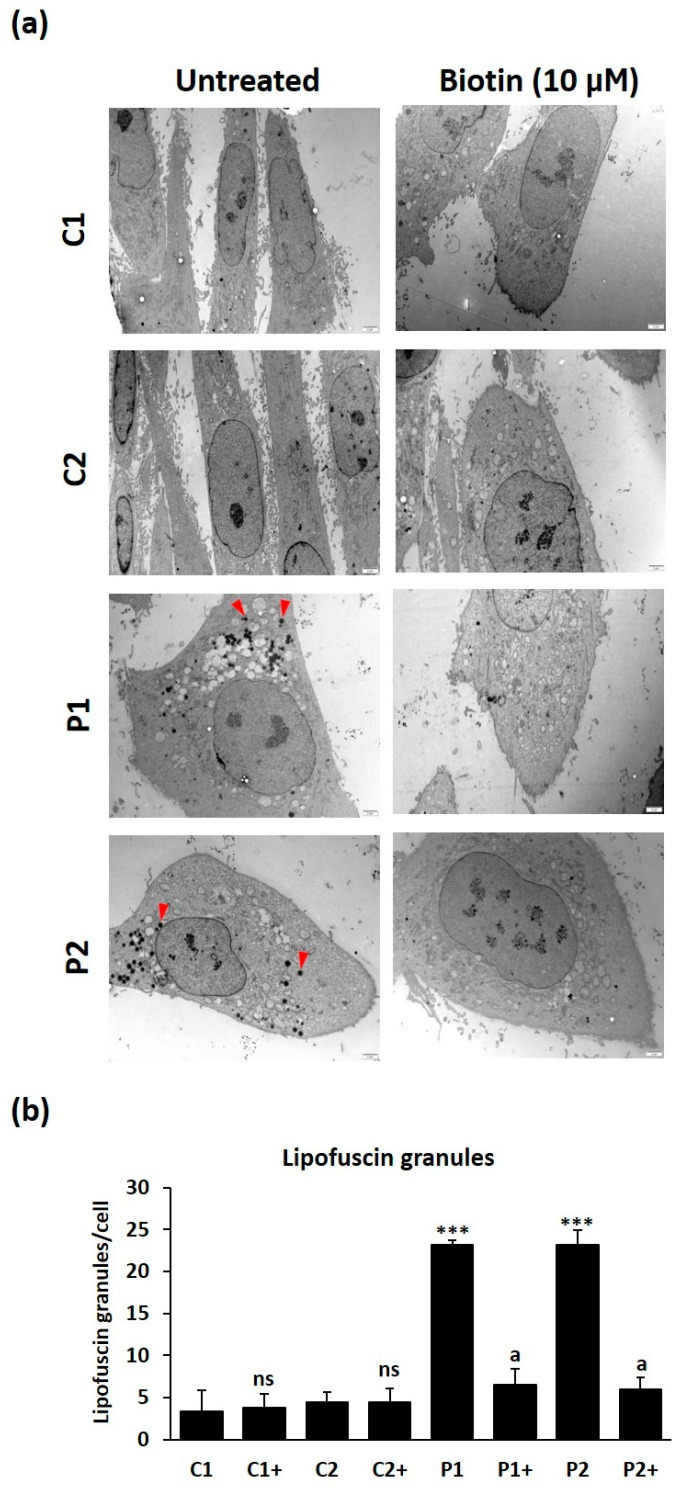
Effect of biotin on lipofuscin accumulation assessed by TEM. (**a**) Representative electron microscopy images of lipofuscin granules (red arrows) of control (C1 and C2) and BPAN (P1 and P2) fibroblasts untreated and treated (+) with 10 μM biotin for one week. Scale bar: 2 μm. (**b**) Quantification of lipofuscin granules per cell. At least 30 images per condition were analyzed. Data represent the mean ± SD of three separate experiments. *** *p* < 0.001 between BPAN and controls cells. ^a^ *p* < 0.01 between the presence and the absence of biotin; ns: not significant.

We next analyzed the ability of biotin supplementation on the prevention of iron/lipofuscin buildup in BPAN fibroblasts. Biotin treatment significantly reduced iron levels in BPAN cells performed by Prussian Blue staining, inductively coupled plasma mass spectrometry (ICP-MS), and labile iron pool (LIP) (Figure 13a–d). Biotin treatment also inhibited the accumulation of lipofuscin in BPAN cell accumulation evaluated by Sudan Black staining (Figure 14a,b). Furthermore, TEM analysis showed that biotin treatment was able to remove lipofuscin-like aggregates in BPAN cells (Figure 15a,b and Figure S3).

### 2.9. Biotin Treatment Prevents Lipid Peroxidation in BPAN Fibroblasts

**Figure 16:**
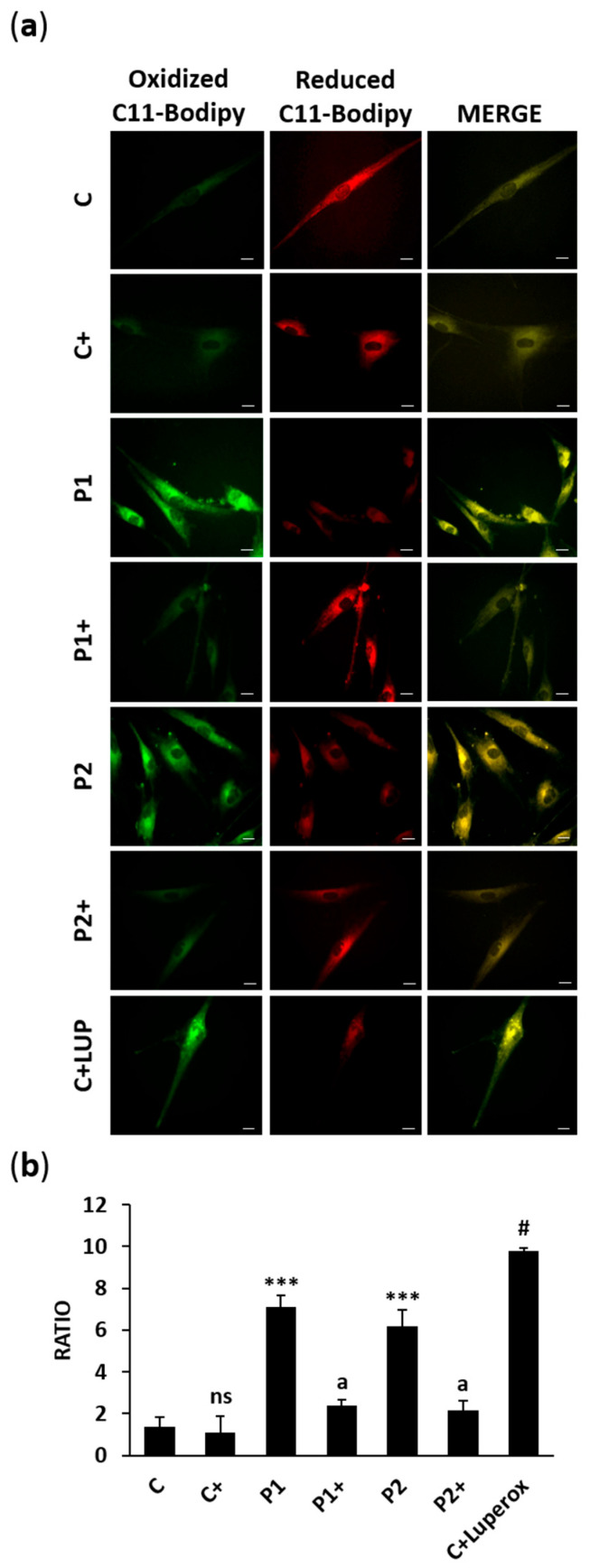
Effect of biotin on lipid peroxidation assessed by C11-Bodipy. (**a**) Representative images of oxidized C11-Bodipy (green) and reduced C11-Bodipy (red) of control (C) and BPAN (P1 and P2) fibroblasts untreated and treated (+) with 10 μM biotin for one week. Control fibroblasts were also treated with 500 μM Luperox (LUP) for 15 min as a positive control of lipid peroxidation. Scale bar: 20 μm. (**b**) Ratio of the oxidized BODIPY-C11 signal to the reduced BODIPY-C11 signal. At least 100 cells per condition were analyzed. Data represent the mean ± SD of three separate experiments. *** *p* < 0.001 between BPAN and control cells. ^a^ *p* < 0.01 between the presence and the absence of biotin. ^#^ *p* < 0.01 between the presence and the absence of Luperox; ns: not significant.

We also performed BODIPY^®^ 581/591 C11 staining to evaluate the biotin supplementation effect on lipid peroxidation levels in BPAN cells (Figure 16a,b). The results showed that biotin treatment was able to significantly prevent lipid peroxidation.

### 2.10. Effect of Biotin Supplementation on BPAN-Induced Neurons (iNs)

Patient-derived fibroblasts serve as valuable cellular models for studying BPAN pathophysiology. However, the most affected cells in NBIA are typically those with high metabolic and energy demands, such as neurons and glial cells [33,34]. Consequently, control and BPAN fibroblasts were reprogrammed into iNs, showing typical neuron-like morphology and positive immunoreactivity for microtubule-associated protein tau (MAPT), a protein found in neuronal axons. Unreprogrammed cells did not show MAPT expression. The results of neuronal purity (number of MAPT+ cells over the total number of cells after reprogramming) and conversion efficiency (number of MAPT+ cells over the total number of cells seeded at the beginning of the experiment) are shown in Figure S4.

**Figure 17:**
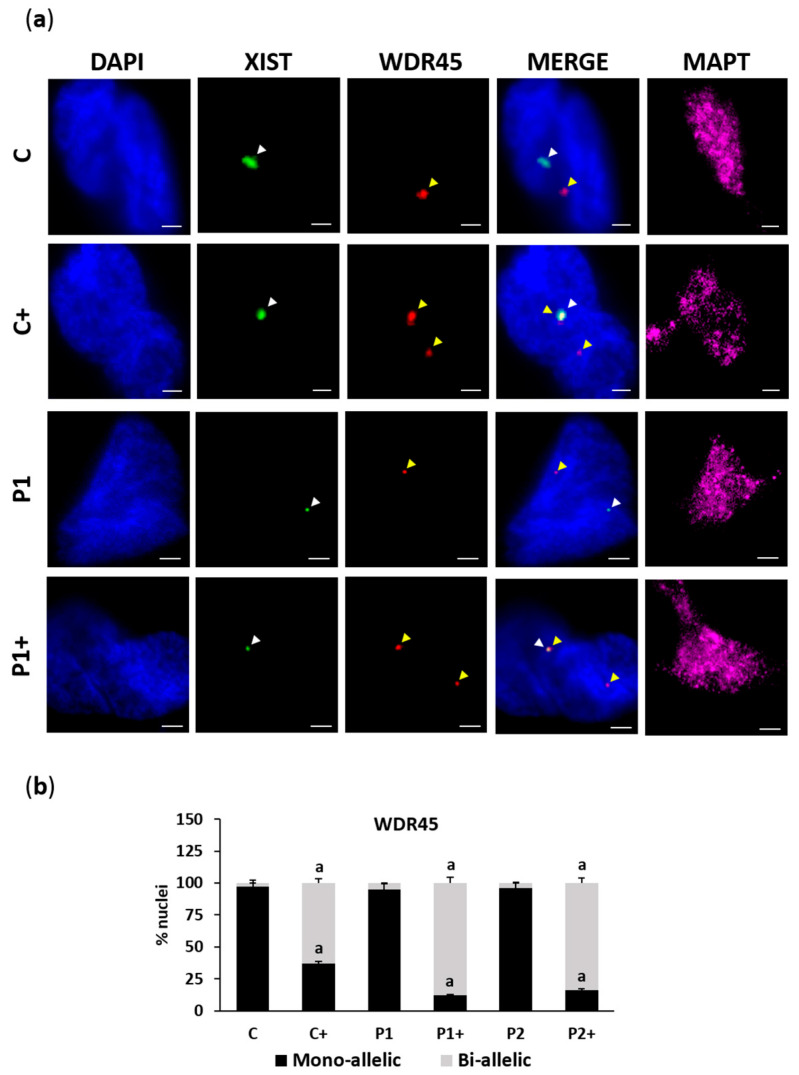
Effect of biotin supplementation on Xi reactivation in iNs. (**a**) Representative images of two-color RNA FISH of control (C) and BPAN (P1) cells, reprogrammed from fibroblasts to iNs, untreated and treated (+) with 10 μM biotin for one week, monitoring the expression of *Xist* (green) and *WDR45* (red). Nuclei were stained with 1 μg/mL DAPI (blue). MAPT (magenta) was used as a neuronal marker. The white arrowheads indicate *Xist* expression, and the yellow arrowheads indicate *WDR45* signals. Scale bar: 20 μm. (**b**) Percentage of monoallelic and biallelic *WDR45* expression. At least 100 neurons per condition were analyzed and only cells with RNA FISH signal were analyzed. Data represent the mean ± SD of three separate experiments. ^a^ *p* < 0.01 between the presence and the absence of biotin.

**Figure 18:**
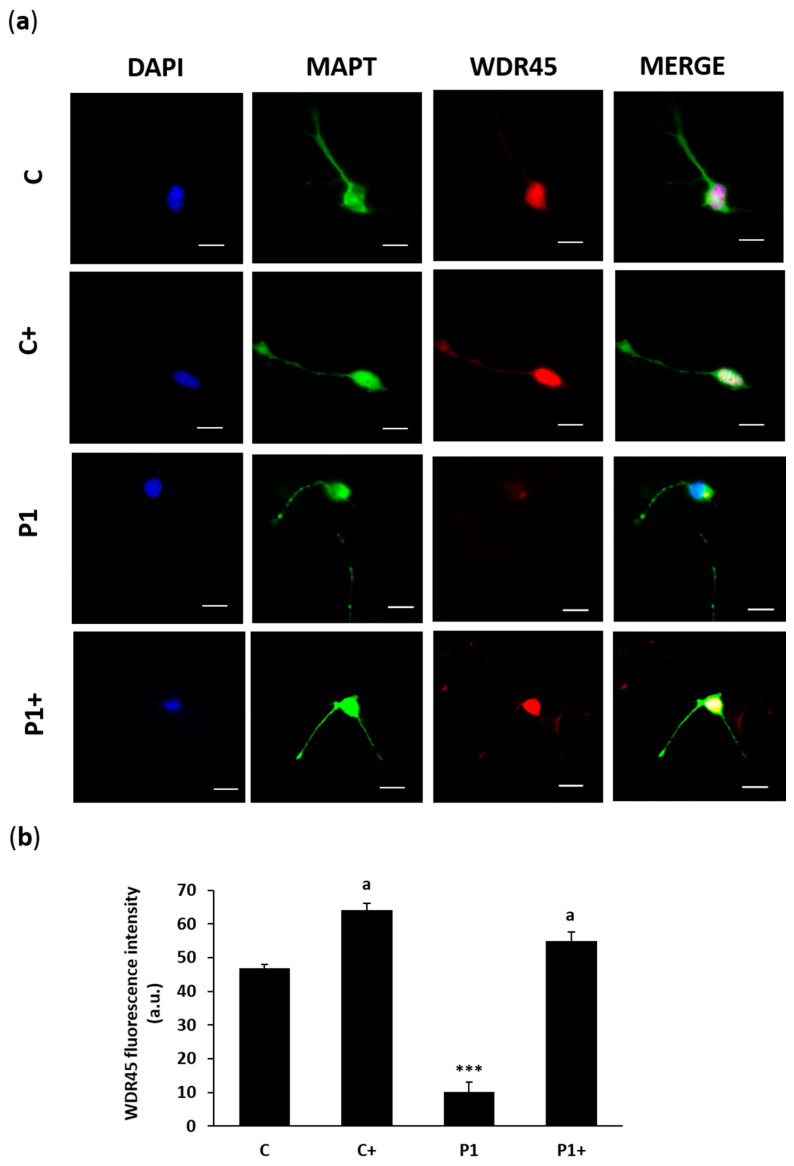
Effect of biotin supplementation on WDR45 protein expression levels in iNs. (**a**) Representative fluorescence images of the anti-WDR45 antibody (red) of control (C) and BPAN (P1) cells, reprogrammed from fibroblasts to iNs, untreated and treated (+) with 10 μM biotin for one week. MAPT (green) was used as a neuronal marker. Nuclei were stained with 1 μg/mL DAPI (blue). Scale bar: 20 μm. (**b**) Quantification of fluorescence intensity of WDR45. At least 100 neurons per condition were analyzed. Data represent the mean ± SD of three separate experiments. *** *p* < 0.001 between BPAN and control cells. ^a^ *p* < 0.01 between the presence and the absence of biotin; a.u.: arbitrary units.

**Figure 19:**
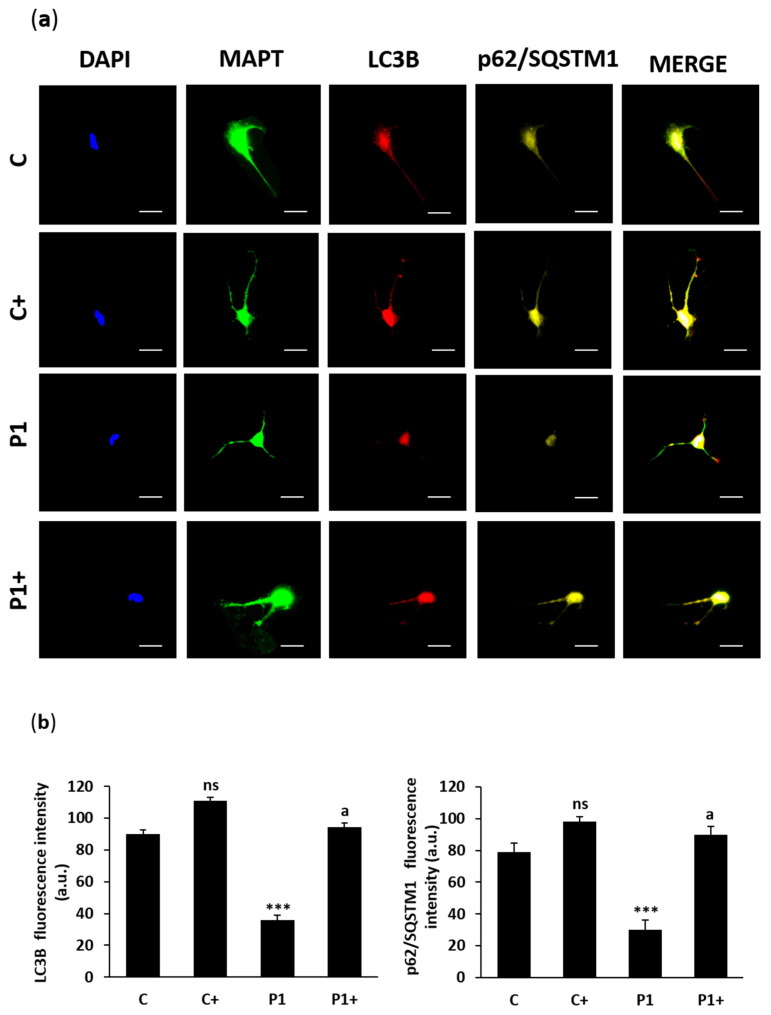
Effect of biotin supplementation on LC3B and p62/SQSTM1 protein expression levels in iNs. (**a**) Representative fluorescence images of anti-LC3B (red) and anti-p62/SQSTM1 (yellow) antibodies of control (C) and BPAN (P1) cells, reprogrammed from fibroblasts to iNs, untreated and treated (+) with 10 μM biotin for one week. MAPT (green) was used as a neuronal marker. Nuclei were stained with 1 μg/mL DAPI (blue). Scale bar: 20 μm. (**b**) Quantification of fluorescence intensity of LC3B and p62/SQSTM1. At least 100 neurons per condition were analyzed. Data represent the mean ± SD of three separate experiments. *** *p* < 0.001 between BPAN and control cells. ^a^ *p* < 0.01 between the presence and the absence of biotin; a.u.: arbitrary units; ns: not significant.

Then, the efficacy of the biotin treatment was evaluated in control and BPAN iNs. The biallelic expression of *WDR45* was performed by RNA FISH assay (Figure 17a,b). In addition, WDR45 protein expression level was measured by immunofluorescence microscopy (Figure 18a,b), as well as LC3B and sequestosome 1 (p62/SQSTM1), two autophagosome markers (Figure 19a,b). Biotin supplementation significantly increased the number of nuclei with biallelic *WDR45* expression, accompanied by enhanced protein expression levels of WDR45 and the autophagosome markers LC3B and p62/SQSTM1 in BPAN cells. These results suggest the activation of the Xi and the restoration of autophagosome formation.

**Figure 20:**
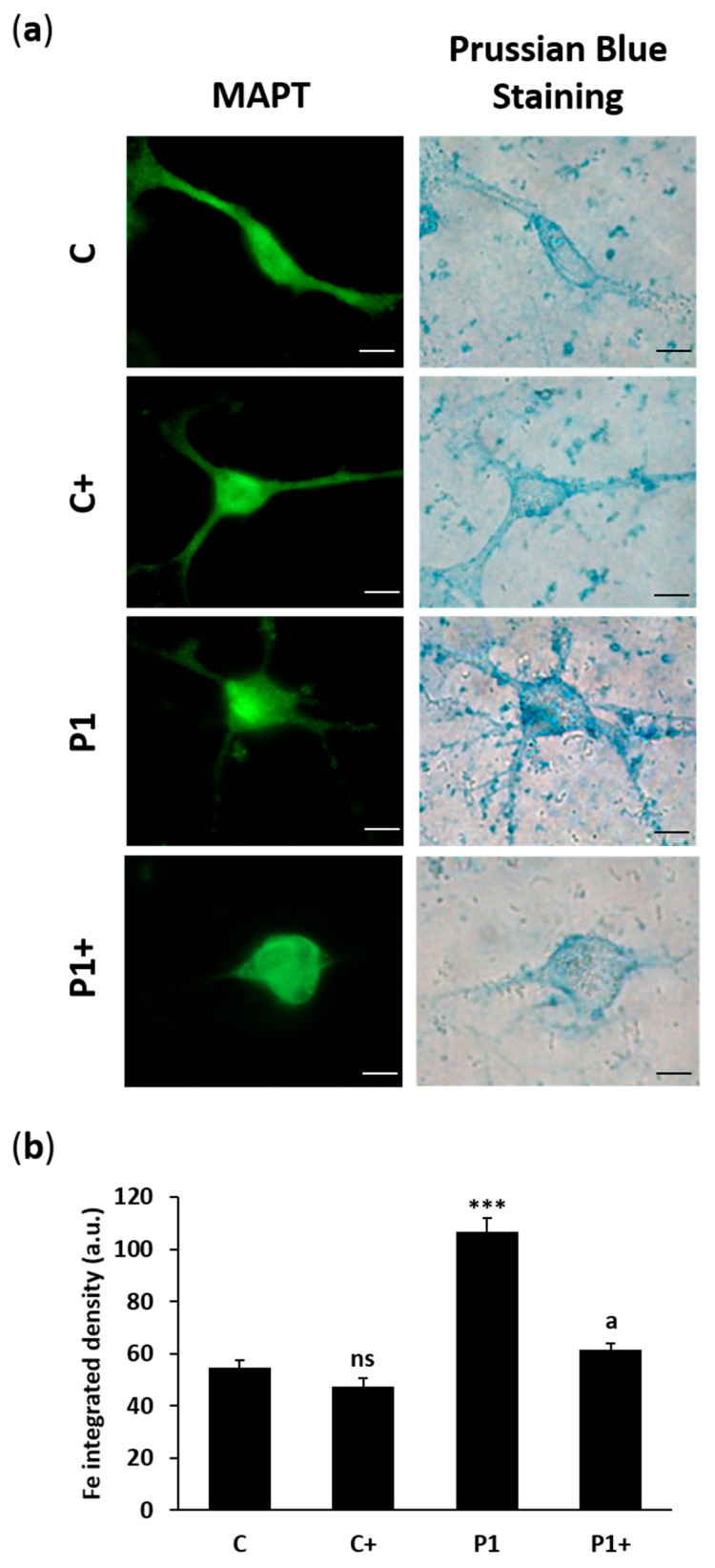
The effect of biotin on iron accumulation in iNs. (**a**) Representative images of Prussian Blue staining of control (C) and BPAN (P1) cells, reprogrammed from fibroblasts to iNs, untreated and treated (+) with 10 μM biotin for one week. MAPT (green) was used as a neuronal marker. Scale bar: 20 μm. (**b**) Quantification of Prussian Blue staining. At least 100 neurons per condition were analyzed. Data represent the mean ± SD of three separate experiments. *** *p* < 0.001 between BPAN and control cells. ^a^ *p* < 0.01 between the presence and the absence of biotin; a.u.: arbitrary units; ns: not significant.

Finally, we evaluated intracellular iron accumulation using Prussian Blue staining (Figure 20a,b). As shown in fibroblasts, BPAN iNs present iron overload, and biotin treatment was able to significantly reduce iron accumulation to control iNs levels.

## 3. Discussion

In a previous study, we showed that *WDR45* mutations induce several cellular pathological alterations including autophagy deficiency, decreased levels of lysosomal proteins and enzymes, impaired mitochondrial bioenergetics and network, iron accumulation, and increased mitochondrial lipid peroxidation [21]. Furthermore, we found that antioxidant supplementation partially improved cell physiopathology; however, autophagy and cell bioenergetics remained affected in BPAN cells [21].

In this work, our objective was to increase the levels of the wild-type allele by reactivating Xi, a strategy that can overcome *WDR45* deficiency and, therefore, correct the mutant phenotype. We found that biotin supplementation trough a mechanism involving histone biotinylation in Xi increased wild-type *WDR45* transcript and protein expression levels (Figure 1) and significantly improved all the pathological consequences of *WDR45* mutations including autophagy/mitophagy deficiency (Figure 6 and Figure 8), mitochondrial bioenergetics failure (Figure 9, Figure 10 and Figure 11), iron/lipofuscin accumulation (Figure 13, Figure 14 and Figure 15), and lipid peroxidation (Figure 16).

X-linked gene expression is equalized between the sexes through the random transcriptional silencing of most genes of one of the two X-chromosomes in females. This X-chromosome inactivation (XCI) is an essential process established during early embryogenesis and maintained afterward in daughter cells throughout prenatal and postnatal life. XCI is triggered by the accumulation of the *Xist* long noncoding RNA (lncRNA) on one of the two X-chromosomes (the future Xi). *Xist* then recruits a series of factors inducing gene silencing in cis [35].

A significant genetic characteristic of BPAN is that the majority of mutations occur de novo, which, when paired with particular clinical features, enables medical professionals to diagnose BPAN [16]. The phenotypic spectrum in BPAN is influenced by somatic mosaicism derived from a de novo postzygotic mutation and, in females, by XCI [36]. Somatic mosaicism describes the presence of two sets of cells in an individual, one with a mutation in *WDR45* and one without. Gonadal mosaicism also occurs in BPAN [37]. For this reason, genotype alone is not a strong predictor of BPAN phenotype, which also depends on several factors: (1) the timing and tissue distribution of somatic mutation during development, (2) the presence of a second X-chromosome, and (3) the pattern of XCI. In females, typically, one X-chromosome is randomly ‘turned off’ in each cell. XCI is widely believed to be random in early female development and to result in a mosaic distribution of cells, approximately half with the paternally derived XCI and half with the maternally derived XCI [38]. However, when one X-chromosome harbors a deleterious allele, XCI can be non-random because selection may favor cells with an active X-chromosome carrying the normal allele, displacing cells with the mutation-bearing chromosome [39]. For this reason, in BPAN, the pattern of XCI may be non-random, thereby influencing the phenotype [7,40,41]. It is possible that somatic mosaicism or favorable XCI characterize asymptomatic or mildly phenotypical female outliers. Somatic mosaicism in males can range from mild to classic BPAN, while non-mosaic cases are typically more severe than classic female cases [37,42].

Considering these characteristics, one strategy of restoring *WDR45* expression in BPAN females consists of the reactivation of the normal copy on the other X-chromosome (Figure 2). However, if this X-chromosome has been inactivated by XCI, this must be reactivated for its therapeutical application. This strategy called ‘Xi reactivation’ involves using epigenetic substances to selectively increase the expression of Xi while reducing the impact on other chromosomes within the same cells [22]. Selective Xi reactivation has several key advantages: first, it is aimed directly at treating the principal cause rather than the secondary effects of X-linked mutations; second, it has the potential to be a strategy for multiple X-linked disorders in females, including BPAN disease, Fragile X, or Rett syndromes; third, Xi reactivation could be applied to every female patient independent of the respective mutation; and lastly, this approach does not require the introduction of viral vectors and the insertion of foreign genetic material in the DNA. Furthermore, because it is wholly epigenetic, it can be temporarily reversed if that becomes necessary [22]. Furthermore, since Xi is strongly suppressed by numerous parallel mechanisms and X-linked gene expression is governed by endogenous transcriptional regulation, it has been postulated that there is little to no risk of gene overexpression due to an imbalance of the X-to-autosome dosage [22,43]. Notably, several mechanisms that regulate the expression of X-linked genes have been proposed [44,45].

In this regard, one key consideration is the timing of Xi reactivation. XCI is known to be essential for early embryonic development. Transmitting a germline *Xist* deletion to daughter embryos causes failed XCI and peri-implantation lethality [46]. Furthermore, a zygotic deletion of *Xist* prior to the onset of XCI is also incompatible with development, though, surprisingly, some female mice survive to birth [47]. Critically, selective Xi reactivation aims to reactivate the Xi in somatic cells, not during embryogenesis, and only long after the Xi is established. Thus, the critical question regarding its safety is whether Xi reactivation could be well tolerated in post-XCI cells. To address this question, studies have systematically deleted *Xist* in various organs of the mouse to assess the degree of Xi reactivation and to investigate morbidity and mortality. Interestingly, mice with conditional post-XCI *Xist* deletion occurring throughout the embryo, in epithelial cells, or in the gut are phenotypically normal with unchanged lifespan in the absence of morbidities [48]. One exception was observed is a deletion of *Xist* in hematopoietic stem cells (HSCs), which resulted in increased mortality in females due to hematologic malignancies [49]. Curiously, however, an *Xist* deletion in B-cells had no effect whatsoever on morbidity or mortality [48]. In a recent study, researchers perturbed the expression of *Xist* in female mice, inducing the reactivation of genes on Xi, including members of the toll-like receptor 7 (TLR7) signaling pathway, in monocyte/macrophages and dendritic and B cells [50]. Consequently, female mice spontaneously developed inflammatory signs typical of lupus, including anti-nucleic acid autoantibodies, increased frequencies of age-associated and germinal center B cells, and the expansion of monocyte/macrophages and dendritic cells. These findings highlight that altered XCI may predispose mice to autoimmunity [50].

Thus, temporal as well spatial factors (the organs in which the Xi is reactivated) are also crucial for safety concerns for Xi reactivation. In this regard, as BPAN disease is a neurodevelopmental disorder, the response of brain cells to Xi reactivation is of particular interest. In one study, mice with a conditional brain *Xist* deletion and partial Xi reactivation were healthy and fertile within a follow-up of up to 2 years [51]. Even when the level of Xi reactivation was increased by combining a brain-specific *Xist* deletion with the DNA methylation inhibitor decitabine for one week, mice demonstrated no impairments in health and life span [52]. Altogether, these studies suggest that post-XCI *Xist* ablation can result in significant Xi reactivation and can be tolerated in the mouse if the exposure to hematopoietic stem cells (HSCs) could be avoided. A therapeutic approach using Xi reactivation will, therefore, depend on targeting relevant affected organs while minimizing exposure to other organs.

Unfortunately, the majority of the research carried out on X-chromosome regulation has been conducted on mice, and from the few studies that have been conducted on human embryos, it appears that their dosage compensation mechanisms are very different [53,54,55]. These findings emphasize the necessity for suitable XCI models at different stages of human development [56].

It has been suggested that autophagy is a crucial mechanism enabling neuronal survival in adult organisms, and that it plays a special role in maintaining the homeostasis of non-dividing cells [57]. Thus, impaired autophagy in neurodevelopmental and neurodegenerative disorders such as BPAN have led to the hypothesis that restoring autophagy could serve as a putative therapeutic strategy to mitigate neurodegeneration [58].

In our work, we found that the biotin supplementation of BPAN iNs increased *WDR45* biallelic expression. Interestingly, one of the WDR45 signals was near the *Xist* signal, suggesting Xi reactivation (Figure 17). These findings were associated with an up-regulation of WDR45 protein expression levels (Figure 18) and a significant increase in autophagosome markers LC3B and p62/SQSTM1 (Figure 19), as well as the prevention of iron accumulation (Figure 20). Altogether, these findings suggest that biotin treatment is also able to correct the mutant phenotype in post-mitotic cells such as iNs, one of the cell types expected to be most affected in BPAN.

The genetic material that makes up nearly every cell in the human body is encoded in roughly two meters of linear DNA [59]. The human genome presents a major organizational challenge because it must be organized into a relatively small nuclear volume while remaining accessible in a coordinated temporal and spatial manner. The solution is achieved by the DNA molecules becoming associated with proteins, predominantly conserved histone proteins, to form a complex macromolecular structure termed chromatin. It is commonly believed that chromatinized DNA eventually folds into stable higher-order (condensed) chromosomal structures. Consequently, decondensed DNA is needed to enable DNA-templated processes like transcription, recombination, replication, and repair. The available data indicate a dynamic genomic architectural landscape where chromatin is constantly changing and transitioning between different states [60]. Thus, chromatin, more than an inert packaging structure, is a dynamic scaffold that can respond to specific signals to regulate DNA accessibility to various components of cellular machinery.

The fundamental unit of chromatin is the nucleosome, which consists of a central histone octamer (two each of histones H2A, H2B, H3, and H4) around which are wound approximately 1.75 left-handed superhelical turns of DNA [61]. These histones are modified by several post-translational modifications (PTMs), often referred to as epigenetic marks, that regulate chromatin structure and, hence, DNA-templated processes.

Histone PTMs have even been suggested to serve as an epigenetic code, in which individual marks carry a unique message [62].

The histone PTM landscape is established, maintained, and readjust by numerous interconnected signaling pathways, which involve enzymes that catalyze the formation of specific types of PTMs (writers), proteins that recognize particular PTMs via specific domains (readers), and enzymes that remove PTMs (erasers). Many of the enzymes involved in histone modification rely on cofactors that intimately link their activity with cellular metabolic states [63,64]. The most common histones PTMs are methylation, phosphorylation, acetylation, and ubiquitination [59]. In addition, histones were also found to be modified by the attachment of biotin, a water-soluble vitamin with an essential role in intermediary metabolism where it acts as a carboxyl carrier in carboxylation reactions (Figure 3, Figure 4 and Figure 5) [65]. Biotinylation is unique among histone modifications in that a metabolic co-factor also functions as a chromatin mark. Biotin is attached to the ɛ-amino group of a strictly conserved lysine in carboxylases through the action of HLCS in a two-step ATP-dependent reaction, generating biotinyl-5′-AMP as an intermediate [66,67,68]. Biotin is recycled from proteolytically degraded carboxylases by BTD, which cleaves biotin from biotinyllysine (biocytin) or small peptides containing biotin [69]. The biotin moiety is covalently bound to the ɛ-amino group of a Lys residue in each of these carboxylases in a domain 60–80 amino acids long [66]. The domain is structurally similar among carboxylases from bacteria to mammals. HLCS catalyzes the transfer of biotin to all of the apocarboxylase substrates [70].

As biotin is a metabolic co-factor that also functions as a chromatin mark, histone biotinylation could act as a metabolic sensor, mediating changes in transcription in response to changes in nutritional status. Biotin modifies tails of histone H2A, H3, and H4 through a covalent attachment of biotin to specific lysine residues catalyzed by HLCS [71]. Biotinylations at histone H4 lysine 8 and lysine 12 have been associated with heterochromatin structures, gene silencing, the mitotic condensation of chromatin, and DNA repair [72,73]. Histone biotinylation is a reversible process, even though debiotinylases have not been well characterized [25]. Dietary supplementation of biotin is required for biotinylation and a deficiency in biotin may have profound effects on chromatin structure [74], although a cultured cell study suggests that biotin is absent in native histones [75]. Because there are many unanswered questions in histone biotinylation, further studies are needed to delineate the significance of histone biotinylation, a direct modification of the histone by a nutrient.

This study presents an important limitation that could be addressed in future research: this study focused on the epigenetic effect of biotin supplementation on WDR45 expression, although its effect on the expression of other genes linked to chromosome X or other metabolic processes was not evaluated.

## 4. Materials and Methods

### 4.1. Reagents

Anti-beta-actin (MyBioSource, MBS448085), Anti-WDR45 (ThermoFisher Scientific, PA5-77801), Anti-LC3B (Cell Signaling Technology, 2775), Anti-p62/SQSTM1 (Abcam, ab91526), Anti-MT-CO2 (Abcam, ab79393), Anti-COX4I1 (Abcam, ab14744), Anti-VDAC1 (Abcam, ab14734), Anti-NDUFS4 (Abcam, ab137064), Anti-NDUFA9 (Abcam, ab14713), Anti-Biotin (ThermoFisher Scientific, 31852), Anti-MAPT (ThermoFisher Scientific, PA5-143582), Anti-HLCS (ThermoFisher Scientific, A304-261A-T), Anti-BTD (Novus Biological, NBP2-97093), Anti-ATP5F1A (Abcam, ab14748).

LysoTracker™ Green DND-26 (ThermoFisher Scientific, L7526), 4′,6-diamidino-2-phenylindole (DAPI) (ThermoFisher Scientific, 62248), Hoechst 33,342 (ThermoFisher Scientific, H1399), CCCP (Sigma-Aldrich, C2759), FCCP (Santa Cruz Biotechnology, sc-203578), rotenone (Santa Cruz Biotechnology, sc-203342), antimycin A (Santa Cruz Biotechnology, sc-202467A), oligomycin (Santa Cruz Biotechnology, sc-203342), Paraformaldehyde (Santa Cruz Biotechnology, sc-253236), Dulbecco’s modified eagle’s medium (DMEM) (ThermoFisher Scientific, 10524684), Penicillin–Streptomycin (ThermoFisher Scientific, 11548876), Trypsine (ThermoFisher Scientific, 15090-046), Mitotracker^TM^ Red CMXRos (ThermoFisher Scientific, M7512), fetal bovine serum (FBS) (Sigma-Aldrich, 12106C), biotin (Santa Cruz Biotechnology, sc-20476), dimethyl sulfoxide (DMSO) (Sigma-Aldrich, 17093), trypan blue (ThermoFisher Scientific, 15250061), Perl’s Prussian Blue (Sigma-Aldrich, 03899), deferiprone (Santa Cruz Biotechnology, sc-211220), Sudan Black (Sigma-Aldrich, 199664), glutaraldehyde 25% aqueous solution (Sigma-Aldrich, G5882), Luperox^®^ DI (Sigma-Aldrich, 168521), BODIPY^®^ 581/591 C11 (ThermoFisher Scientific, D3861), Donkey serum (Sigma-Aldrich, D9663), histone extraction kit (Abcam, ab113476), Premo™ Autophagy Tandem Sensor RFP-GFP-LC3B kit (ThermoFisher Scientific, P36239), mitophagy detection kit (Dojindo Molecular Technology, MD01).

### 4.2. Patient Cell Culture

We obtained primary skin fibroblasts from two healthy individuals matched for age and sex (controls 1 and 2), and from two BPAN patients (P1 and P2) with clinical and radiological evidence compatible with BPAN. P1 is a 7-year-old female with a heterozygous frameshift mutation c.400C>T (p. Arg134*), which results in a truncated WDR45 protein [41]. P2 is a 14-year-old female carrying a heterozygous c.182A>C mutation, leading to an amino acid substitution of asparagine to threonine at position 61 (p. Asn61Thr). In silico predictions using SIFT and PolyPhen-2 algorithms suggest that this p. Asn61Thr mutation is a deletion [76]. Additionally, a different pathogenic variant at the same amino acid position (p. Asn61Lys) has been previously reported [36].

Fibroblasts derived from the patients and controls were cultured at 37 °C and 5% CO_2_ in DMEM supplemented with a 1% antibiotic Penicillin–Streptomycin solution and 10% FBS. In the medium, the concentration of ferric nitrate was 0.1 mg/L. All the experiments were performed with fibroblasts on a passage number lower than 10. Cells were treated with 10 μM of biotin, as minimum positive concentration in both cell lines, for 7 days based on previous studies from our group [21].

### 4.3. Immunoblotting

Western blot assay was performed following standard procedures. After protein transfer to nitrocellulose membranes (Bio-Rad, Hercules, CA, USA), the membranes were incubated overnight at 4 °C with primary antibodies at proper dilution range (1:100–1:1000), and then with the corresponding secondary antibody coupled to horseradish peroxidase diluted 1:2500. Protein bands were identified by ChemiDoc™ MP Imaging System (Bio-Rad, Hercules, CA, USA) using the Immun Star HRP substrate kit (Biorad Laboratories Inc., Hercules, CA, USA).

In cases where different proteins of interest did not share overlapping molecular weights, the membranes were reincubated with additional antibodies or cut into sections for detection with different antibodies. Results were normalized to the housekeeping protein actin and analyzed using ImageLab™ version 5.0 software (Bio-Rad, Hercules, CA, USA).

### 4.4. Real-Time Quantitative PCR

mRNA extracts were analyzed using real-time quantitative PCR (RT-qPCR) to assess WDR45 gene expression fibroblasts. Standard extraction techniques were used to isolate total mRNA. Gene expression levels were measured using SYBR Green methods. WDR45 gene was amplified using the specific primers 5′-TTTACGGTTCCGGGTACTGG-3′ (forward) and 5′-AATTTCAACCTCCACCAGCG-3′ (reverse), generating a 98-nucleotide product. Actin was used as a housekeeping control with the following primers: 5′-AGAGCTACGAGCTGCCTGAC-3′ (forward) and 5′-AGCACTGTGTTGGCGTACAG-3′ (reverse). The primers were designed using the online tool Primer3 (https://primer3.ut.ee/).

### 4.5. Immunofluorescence Microscopy

The immunofluorescence protocol was previously described by our research group [77]. Fibroblasts were seeded on glass cover slips (Menzel-Gläser, ThermoFisher Scientific, Waltham, MA, USA), whereas iNs were plated on a 4-well μ-Slide (Ibidi Inc., Martinsried, Germany). Images and Pearson correlation coefficients were obtained using a DeltaVision system (Applied Precision; Issaquah, WA, USA) with an Olympus IX-71 fluorescence microscope.

All experiments were carried out in triplicate for each condition and repeated at least three times. In each set of experiments, images were recorded with identical microscope settings. Quantitation was performed using the Fiji-ImageJ software version 2.9.0. in raw uncompressed images. The assay was validated with positive or negative controls.

### 4.6. RNA FISH

RNA FISH experiments were executed as previously described [78]. The labeled probes were obtained from Metabion International AG (see Table S1). Cells were visualized on a DeltaVision system (Applied Precision; Issaquah, WA, USA) with an Olympus IX-71 fluorescence microscope with a 100× objective; 100 independent cells were counted and evaluated. Only cells with a detectable RNA FISH signal were analyzed by Fiji-ImageJ software version 2.9.0.

### 4.7. Histone Biotinylation

Histone extraction was performed using a histone extraction kit (Abcam, ab113476) following the manufacturer’s instructions. Histone protein concentrations were quantified by Lowry’s method using DC Protein Assay (Bio-Rad, 5000111) and read by a POLARstar OMEGA Microplate Reader (BMG Labtech, Ortenberg, Germany) at 750 nm. A total of 10 µg of each sample was loaded per lane onto a 15% Tris/glycine polyacrylamide gel. Gels were run for 2 h and incubated with anti-biotin (ThermoFisher Scientific, 31852; 1:1000). Ponceau Red staining was used to evaluate protein loading and normalize the levels of anti-biotin for each sample. The intensity of the bands was measured using ImageLab™ version 5.0 software (Bio-Rad, Hercules, CA, USA).

Alternatively, histone biotinylation was assessed by immunofluorescence microscopy.

### 4.8. Tandem Sensor RFP- GFP-LC3B

Premo™ Autophagy Tandem Sensor RFP-GFP-LC3B Kit (Thermo Fisher Scientific, P36239) was used to measure autophagic flux in accordance with the manufacturer’s instructions. The cells were transduced with 40 particles per cell for 16 h after being treated with 10 μL of BacMam reagents. Following incubation, the cells were left in medium for 48 h to bring the expression levels of autophagy markers back to normal. Cells were treated with 90 μM Cq (component B) for 16 h and nuclei were labeled by Hoechst 33342. Images were taken using DeltaVision system (Applied Precision; Issaquah, WA, USA) with an Olympus IX-71 fluorescent microscope with a 40× objective and analyzed by Fiji-ImageJ software version 2.9.0. RFP showed autolysosome production and GFP showed LC3B positive autophagosomes. Red fluorescence indicates acidic pH autolysosomes, whereas green and red fluorescence colocalization (GFP + RFP) indicates neutral pH autophagosomes.

### 4.9. Lysotracker Staining

Fibroblasts acidic compartments, such as lysosomes and autolysosomes, were stained using the acidotropic dye LysoTracker™ Green DND-26 at 75 nM for an hour. The samples were observed using DeltaVision system (Applied Precision; Issaqua, WA, USA) with an Olympus IX-71 fluorescent microscope with a 40× objective. The fluorescence intensity of Lysotracker was counted by Fiji-ImageJ software version 2.9.0.

### 4.10. Mitophagy Assay

Mitochondrial mitophagy was tested using a mitophagy detection kit (Dojindo Molecular Technology, MD01) following the manufacturer’s methods as previously described [79]. Mitophagy Dye was used for mitophagy detection and Lyso Dye as a staining agent for lysosomes, allowing us to quantify the damaged mitochondria fusing to the lysosomes. Images were visualized using DeltaVision system (Applied Precision; Issaqua, WA, USA) with an Olympus IX-71 fluorescent microscope with a 40× objective. The colocalization of Mitophagy Dye and Lyso Dye was measured using the Pearson correlation coefficient with Fiji-ImageJ software version 2.9.0.

### 4.11. Bioenergetic and Oxidative Stress Analysis

Mito-stress test assay was used to determinate mitochondrial respiration function by XF24 extracellular flux analyzer (Seahorse Bioscience, Billerica, MA, USA) according to the manufacturer’s instructions. The day before the assay, approximately 1.5 × 10^4^ cells/well (minimum five wells/experimented condition) were seeded in a Seahorse XF microplate (Agilent Technologies, 100777-004) and the Seahorse XF Analyzer was prepared by hydrating the sensor cartridge. Once the cells were seeded, they were incubated overnight in a CO_2_; incubator. The next day, the growth medium was replaced with Seahorse XF assay medium (Agilent Technologies, 102353-100), supplemented with 10 mM D-glucose, 1 mM L-glutamine, and 1 mM sodium pyruvate to support cellular metabolism. Control and BPAN fibroblasts were then incubated in a non-CO_2_ incubator for about an hour, allowing them to acclimate to the conditions of the assay. Different metabolic inhibitors or activators, such as oligomycin, FCCP, and rotenone/antimycin, were sequentially injected into the wells during the assay. The parameters quantified were basal and maximal respiration, spare respiratory capacity, and ATP production by calculating the oxygen consumption rate (OCR; pmol O_2_/min). The results were normalized to cell number by cell counting using the BioTek^TM^ Cytation 1 Cell Imaging Multi-Mode Reader (Biotek, Winooski, VT, USA).

### 4.12. Analysis of the Mitochondrial Network by Mitotracker Staining

Fibroblasts were incubated at 37 °C with MitoTracker Red CMXRos (100 nM, 45 min), a fluorescent dye sensitive to mitochondrial membrane potential. Images were captured using DeltaVision system (Applied Precision; Issaqua, WA, USA) with an Olympus IX-71 fluorescent microscope with a 40× objective. Cells fluorescence intensity and the percentage of rounded/tubular mitochondria was quantified using Fiji-ImageJ software version 2.9.0. Rounded mitochondria were defined as 0.2–0.5 µm^2^ and tubular mitochondria as >0.5 µm^2^. The mitochondrion area was measured considering the major and minor axes of the organelle.

### 4.13. Cell Viability Assay

Cell viability was performed using trypan blue exclusion method [80]. Trypan blue was used to count cells using the Countess™ 3 Automated Cell Counter (Invitrogen™, Eugene, OR, USA) at different times.

### 4.14. Prussian Blue Staining and ICP-MS

Iron accumulation was measured in fibroblasts and iNs using Prussian Blue staining as previously described by our research group [77]. P1 cells treated with 100 μM deferiprone were used as a negative control of the technique. Images were obtained using an Axio Vert A1 microscope (Zeiss, Oberkochen, Germany) with a 20× objective and analyzed by Fiji-ImageJ software version 2.9.0.

In fibroblasts, iron content was also evaluated in cell extracts (obtained by acid digestion with HNO_3)_ using ICP-MS [81]. This assay was executed with an Agilent 7800 spectrometer (Agilent Technologies, Santa Clara, CA, USA).

### 4.15. Labile Iron Pool (LIP) Determination

Control and BPAN fibroblasts were seeded in 96-well plates and incubated in a medium containing 1 mg/mL bovine serum albumin (BSA) and 0.25 µM Calcein-AM (37 °C, 15 min). After the incubation, cells were washed twice with hank’s balanced salt solution (HBSS) and then incubated in hank’s balanced salt solution (HBSS) supplemented with 10 mM glucose for 10 min. Basal fluorescence was evaluated by POLARstar OMEGA Microplate Reader (BMG Labtech, Ortenberg, Germany) at 485 nm (excitation) and 535 nm (emission). Afterward, cells were treated with 0.1 mM Salicylaldehyde Isonicotinoyl Hydrazone (SIH), an iron-specific chelator, for 15 min. Fluorescence was monitored during chelator incubation, and once a plateau was reached, the LIP value was determined. All fluorescence data were normalized to protein concentration. P1 cells treated with 100 μM deferiprone were used as a negative control of the technique.

### 4.16. Lipofuscin Accumulation

Sudan Black B (SBB) staining was used to determinate lipofuscin accumulation in control and BPAN fibroblasts according to published protocols [82]. Cells were visualized using an Axio Vert A1 microscope (Zeiss, Oberkochen, Germany) with a 20× objective. The intensity of Sudan Black staining was measured by Fiji-ImageJ software version 2.9.0.

### 4.17. TEM Analysis

Fibroblasts were plated on 8-well Permanox chamber slides (Nunc, ThermoFisher Scientific, Waltham, MA, USA). First, cells were fixed in tempered 3.5% glutaraldehyde in 0.1 M phosphate buffer (PB) (37 °C, 5 min or, 4 °C, 55 min) and, afterwards, were postfixed in 2% OsO_4_ for 1 h at room temperature, washed, dehydrated, and integrated in Durcupan™ ACM (Sigma-Aldrich, 44611). Cells were cut in 70 nm sections with a diamond knife and analyzed by transmission electron microscope (TEM) (FEI Tecnai G2 Spirit BioTwin) with a Xarosa (20-Megapixel resolution) digital camera using Radius image acquisition software version 2.1. (EMSIS GmbH, Münster, Germany).

### 4.18. Lipid Peroxidation

Fibroblasts were incubated at 37 °C with 5 µM BODIPY^®^ 581/591 C11 (ThermoFisher Scientific, D3861) for 30 min to evaluate cell lipid peroxidation. Luperox^®^ was used as a positive control in control cells at 500 μM. Images were taken using an Axio Vert A1 fluorescence microscope (Zeiss, Oberkochen, Germany) with a 20× objective. The results were analyzed by Fiji-ImageJ software version 2.9.0.

### 4.19. Direct Reprograming

Direct reprogramming [83] was performed to generate control and BPAN iNs. Fibroblasts were plated on 4-well μ-Slide Ibidi plates (Ibidi Inc., Martinsried, Germany) and cultured in a DMEM Glutamax medium (ThermoFisher Scientific, 10566016) supplemented with 1% Penicillin–Streptomycin solution and 10% FBS. The next day, fibroblasts were infected with one single lentiviral vector containing two shRNAs against the REST complex and two neural lineage-specific transcription factors, *Achaete-Scute Family BHLH Transcription Factor 1* (*ASCL1*) *and POU class 3 homeobox 2* (*BRN2*), which were obtained as previously described [84]. The multiplicity of infection (MOI) was 30. Plasmids were a gift from Malin Parmar (Developmental and Regenerative Neurobiology, Lund University, Sweden). Twenty-four hours after infection, cell medium was substituted with fresh medium. Forty-eight hours later, cell medium was replaced with supplemented NDiff227 as described before [83]. Half of medium was refreshed every 2–3 days. Eighteen days post-infection, medium was switched with NDiff227 supplemented only with growth factors. Conversion efficiency and neuronal purity were quantified at day twenty-seven post-infection considering MAPT+ cells as iNs. Images were obtained from CellDiscoverer7 microscope (Zeiss, Oberkochen, Germany) and analyzed by Fiji-ImageJ software version 2.9.0.

### 4.20. Statistical Analysis

Statistical analysis was carried out as described previously by our research group [85]. If there were few events (n < 30), we used non-parametric statistics that do not have any distributional assumption [86] and compared multiple groups using a Kruskal–Wallis test. We performed parametric tests when the number of events was higher (n > 30) and compared multiple groups using a one-way ANOVA. Statistical analyses were executed using the GraphPad Prism 9.2 (GraphPad Software, San Diego, CA, USA). The results are shown as the mean ± SD values or as representative of at least three independent experiments. *p*-values of less than 0.05 were considered significant.

## 5. Conclusions

Our study showed that biotin supplementation enhanced histone biotynilation in the confined region of chromatin, which colocalizes with *Xist* transcripts that permitted chromosome Xi’s *WDR45* allele transcription. Thus, up-regulating wild-type *WDR45* transcripts and protein expression levels reversed all physiopathological changes in BPAN cells (autophagy deficit, mitochondrial dysfunction, iron/lipofuscin accumulation, and lipid peroxidation). Therefore, the reactivation of chromosome Xi using epigenetic modulators may be a viable treatment strategy for BPAN.

If successful in BPAN, another interesting aspect of this Xi reactivation strategy is that it can be adapted to other heterozygous X-linked disorders, including Rett syndrome [22], cyclin-dependent kinase-like 5 (CDKL5) deficiency disorder [19], Fragile X syndrome [9], and many others [24].

## Data Availability

All data supporting the findings of this study are available within the paper and its Supplementary Information. The datasets used and analyzed during the current study are available from the corresponding authors on reasonable request.

## Supplementary Materials

The following supporting information can be downloaded at: https://www.mdpi.com/article/10.3390/ijms26031315/s1.

## Abbreviations

The following abbreviations are used in this manuscript:

ATP5F1A	ATP-synthase F1 subunit 1 alpha
BPAN	beta-propeller-protein-associated neurodegeneration
BTD	biotinidase
CCCP	carbonyl cyanide m-chlorophenylhydrazone
COX4I1	cytochrome c oxidase subunit 4I1
Cq	chloroquine
DAPI	4′,6-diamidino-2-phenylindole
DMEM	Dulbecco’s modified eagle’s medium
ER	endoplasmic reticulum
FBS	fetal bovine serum
FCCP	carbonyl cyanide 4-(trifluoromethoxy) phenylhydrazone
FISH	fluorescence in situ hybridization
HLCS	holocarboxylase synthetase
ICP-MS	inductively coupled plasma mass spectrometry
iNs	induced neurons
KO	knockout
LC3B	microtubule-associated protein 1 light chain 3 beta
MAPT	microtubule-associated protein tau
MT-CO2	mitochondrially encoded cytochrome c oxidase II
NBIA	neurodegeneration with brain iron accumulation
NDUFA9	NADH:ubiquinone oxidoreductase subunit A9
NDUFS4	NADH:ubiquinone oxidoreductase subunit S4
PTMs	post-translational modifications
p62/SQSTM1	sequestosome 1
TEM	transmission electron microscope
VDAC1	voltage dependent anion channel 1
WDR45	WD repeat domain 45
XCI	X-chromosome inactivation
Xi	inactive chromosome X
Xist	X-inactive-specific transcript

## References

[1] Ganz T. (2013). Systemic iron homeostasis. Physiol. Rev..

[2] Salvador G.A. (2010). Iron in neuronal function and dysfunction. Biofactors.

[3] Uttara B., Singh A.V., Zamboni P., Mahajan R.T. (2009). Oxidative stress and neurodegenerative diseases: A review of upstream and downstream antioxidant therapeutic options. Curr. Neuropharmacol..

[4] Gregory A., Hayflick S., Adam M.P., Ardinger H.H., Pagon R.A., Wallace S.E., Bean L.J.H., Mirzaa G., Amemiya A. (1993). Neurodegeneration with Brain Iron Accumulation Disorders Overview. GeneReviews^®^ [Internet].

[5] Stanga D., Zhao Q., Milev M.P., Saint-Dic D., Jimenez-Mallebrera C., Sacher M. (2019). TRAPPC11 functions in autophagy by recruiting ATG2B-WIPI4/WDR45 to preautophagosomal membranes. Traffic.

[6] Wan H., Wang Q., Chen X., Zeng Q., Shao Y., Fang H., Liao X., Li H.S., Liu M.G., Xu T.L. (2020). WDR45 contributes to neurodegeneration through regulation of ER homeostasis and neuronal death. Autophagy.

[7] Haack T.B., Hogarth P., Gregory A., Prokisch H., Hayflick S.J. (2013). BPAN: The only X-linked dominant NBIA disorder. Int. Rev. Neurobiol..

[8] Saitsu H., Nishimura T., Muramatsu K., Kodera H., Kumada S., Sugai K., Kasai-Yoshida E., Sawaura N., Nishida H., Hoshino A. (2013). De novo mutations in the autophagy gene WDR45 cause static encephalopathy of childhood with neurodegeneration in adulthood. Nat. Genet..

[9] Cong Y., So V., Tijssen M.A.J., Verbeek D.S., Reggiori F., Mauthe M. (2021). WDR45, one gene associated with multiple neurodevelopmental disorders. Autophagy.

[10] Klionsky D.J., Emr S.D. (2000). Autophagy as a regulated pathway of cellular degradation. Science.

[11] Lee J.H., Nam S.O., Kim E.K., Shin J.H., Oh S.H., Ryu D., Lee H.E., Mun J.Y. (2021). Autophagic defects observed in fibroblasts from a patient with beta-propeller protein-associated neurodegeneration. Am. J. Med. Genet. Part A.

[12] Zhao Y.G., Sun L., Miao G., Ji C., Zhao H., Sun H., Miao L., Yoshii S.R., Mizushima N., Wang X. (2015). The autophagy gene Wdr45/Wipi4 regulates learning and memory function and axonal homeostasis. Autophagy.

[13] Ingrassia R., Memo M., Garavaglia B. (2017). Ferrous Iron Up-regulation in Fibroblasts of Patients with Beta Propeller Protein-Associated Neurodegeneration (BPAN). Front. Genet..

[14] Seibler P., Burbulla L.F., Dulovic M., Zittel S., Heine J., Schmidt T., Rudolph F., Westenberger A., Rakovic A., Munchau A. (2018). Iron overload is accompanied by mitochondrial and lysosomal dysfunction in WDR45 mutant cells. Brain.

[15] Adang L.A., Pizzino A., Malhotra A., Dubbs H., Williams C., Sherbini O., Anttonen A.K., Lesca G., Linnankivi T., Laurencin C. (2020). Phenotypic and Imaging Spectrum Associated With WDR45. Pediatr. Neurol..

[16] Wilson J.L., Gregory A., Kurian M.A., Bushlin I., Mochel F., Emrick L., Adang L., Group B.G.C.A., Hogarth P., Hayflick S.J. (2021). Consensus clinical management guideline for beta-propeller protein-associated neurodegeneration. Dev. Med. Child Neurol..

[17] Long M., Abdeen N., Geraghty M.T., Hogarth P., Hayflick S., Venkateswaran S. (2015). Novel WDR45 Mutation and Pathognomonic BPAN Imaging in a Young Female With Mild Cognitive Delay. Pediatrics.

[18] Abidi A., Mignon-Ravix C., Cacciagli P., Girard N., Milh M., Villard L. (2016). Early-onset epileptic encephalopathy as the initial clinical presentation of WDR45 deletion in a male patient. Eur. J. Hum. Genet..

[19] Lee J.H., Yun J.Y., Gregory A., Hogarth P., Hayflick S.J. (2020). Brain MRI Pattern Recognition in Neurodegeneration With Brain Iron Accumulation. Front. Neurol..

[20] Aring L., Choi E.K., Kopera H., Lanigan T., Iwase S., Klionsky D.J., Seo Y.A. (2022). A neurodegeneration gene, WDR45, links impaired ferritinophagy to iron accumulation. J. Neurochem..

[21] Suárez-Carrillo A., Álvarez-Córdoba M., Romero-González A., Talaverón-Rey M., Povea-Cabello S., Cilleros-Holgado P., Piñero-Pérez R., Reche-López D., Gómez-Fernández D., Romero-Domínguez J.M. (2023). Antioxidants Prevent Iron Accumulation and Lipid Peroxidation, but Do Not Correct Autophagy Dysfunction or Mitochondrial Bioenergetics in Cellular Models of BPAN. Int. J. Mol. Sci..

[22] Grimm N.B., Lee J.T. (2022). Selective Xi reactivation and alternative methods to restore MECP2 function in Rett syndrome. Trends Genet..

[23] Ohhata T., Senner C.E., Hemberger M., Wutz A. (2011). Lineage-specific function of the noncoding Tsix RNA for Xist repression and Xi reactivation in mice. Genes Dev..

[24] Migeon B.R. (2020). X-linked diseases: Susceptible females. Genet. Med..

[25] Hassan Y.I., Zempleni J. (2006). Epigenetic regulation of chromatin structure and gene function by biotin. J. Nutr..

[26] Hymes J., Fleischhauer K., Wolf B. (1995). Biotinylation of histones by human serum biotinidase: Assessment of biotinyl-transferase activity in sera from normal individuals and children with biotinidase deficiency. Biochem. Mol. Med..

[27] Lee D.Y., Hayes J.J., Pruss D., Wolffe A.P. (1993). A positive role for histone acetylation in transcription factor access to nucleosomal DNA. Cell.

[28] Pham A.D., Sauer F. (2000). Ubiquitin-activating/conjugating activity of TAFII250, a mediator of activation of gene expression in *Drosophila*. Science.

[29] Sommerville J., Baird J., Turner B.M. (1993). Histone H4 acetylation and transcription in amphibian chromatin. J. Cell. Biol..

[30] Kothapalli N., Camporeale G., Kueh A., Chew Y.C., Oommen A.M., Griffin J.B., Zempleni J. (2005). Biological functions of biotinylated histones. J. Nutr. Biochem..

[31] Wang S., Long H., Hou L., Feng B., Ma Z., Wu Y., Zeng Y., Cai J., Zhang D.W., Zhao G. (2023). The mitophagy pathway and its implications in human diseases. Signal Transduct. Target. Ther..

[32] Noguchi M., Hirata N., Tanaka T., Suizu F., Nakajima H., Chiorini J.A. (2020). Autophagy as a modulator of cell death machinery. Cell Death Dis..

[33] Ismail F.Y., Mitoma H., Fatemi A. (2018). Metabolic ataxias. Handb. Clin. Neurol..

[34] Molnar M.J., Kovacs G.G. (2017). Mitochondrial diseases. Handb. Clin. Neurol..

[35] Loda A., Collombet S., Heard E. (2022). Gene regulation in time and space during X-chromosome inactivation. Nat. Rev. Mol. Cell Biol..

[36] Haack T.B., Hogarth P., Kruer M.C., Gregory A., Wieland T., Schwarzmayr T., Graf E., Sanford L., Meyer E., Kara E. (2012). Exome sequencing reveals de novo WDR45 mutations causing a phenotypically distinct, X-linked dominant form of NBIA. Am. J. Hum. Genet..

[37] Zarate Y.A., Jones J.R., Jones M.A., Millan F., Juusola J., Vertino-Bell A., Schaefer G.B., Kruer M.C. (2016). Lessons from a pair of siblings with BPAN. Eur. J. Hum. Genet..

[38] Puck J.M., Willard H.F. (1998). X inactivation in females with X-linked disease. N. Engl. J. Med..

[39] Van den Veyver I.B. (2001). Skewed X inactivation in X-linked disorders. Semin. Reprod. Med..

[40] Fieremans N., Van Esch H., Holvoet M., Van Goethem G., Devriendt K., Rosello M., Mayo S., Martinez F., Jhangiani S., Muzny D.M. (2016). Identification of Intellectual Disability Genes in Female Patients with a Skewed X-Inactivation Pattern. Hum. Mutat..

[41] Hayflick S.J., Kruer M.C., Gregory A., Haack T.B., Kurian M.A., Houlden H.H., Anderson J., Boddaert N., Sanford L., Harik S.I. (2013). beta-Propeller protein-associated neurodegeneration: A new X-linked dominant disorder with brain iron accumulation. Brain.

[42] Nakashima M., Takano K., Tsuyusaki Y., Yoshitomi S., Shimono M., Aoki Y., Kato M., Aida N., Mizuguchi T., Miyatake S. (2016). WDR45 mutations in three male patients with West syndrome. J. Hum. Genet..

[43] Bhatnagar S., Zhu X., Ou J., Lin L., Chamberlain L., Zhu L.J., Wajapeyee N., Green M.R. (2014). Genetic and pharmacological reactivation of the mammalian inactive X chromosome. Proc. Natl. Acad. Sci. USA.

[44] Deng X., Hiatt J.B., Nguyen D.K., Ercan S., Sturgill D., Hillier L.W., Schlesinger F., Davis C.A., Reinke V.J., Gingeras T.R. (2011). Evidence for compensatory upregulation of expressed X-linked genes in mammals, *Caenorhabditis elegans* and *Drosophila melanogaster*. Nat. Genet..

[45] Nguyen D.K., Disteche C.M. (2006). Dosage compensation of the active X chromosome in mammals. Nat. Genet..

[46] Marahrens Y., Panning B., Dausman J., Strauss W., Jaenisch R. (1997). Xist-deficient mice are defective in dosage compensation but not spermatogenesis. Genes Dev..

[47] Yang L., Kirby J.E., Sunwoo H., Lee J.T. (2016). Female mice lacking Xist RNA show partial dosage compensation and survive to term. Genes Dev..

[48] Yang L., Yildirim E., Kirby J.E., Press W., Lee J.T. (2020). Widespread organ tolerance to Xist loss and X reactivation except under chronic stress in the gut. Proc. Natl. Acad. Sci. USA.

[49] Yildirim E., Kirby J.E., Brown D.E., Mercier F.E., Sadreyev R.I., Scadden D.T., Lee J.T. (2013). Xist RNA is a potent suppressor of hematologic cancer in mice. Cell.

[50] Huret C., Ferraye L., David A., Mohamed M., Valentin N., Charlotte F., Savignac M., Goodhardt M., Guery J.C., Rougeulle C. (2024). Altered X-chromosome inactivation predisposes to autoimmunity. Sci. Adv..

[51] Adrianse R.L., Smith K., Gatbonton-Schwager T., Sripathy S.P., Lao U., Foss E.J., Boers R.G., Boers J.B., Gribnau J., Bedalov A. (2018). Perturbed maintenance of transcriptional repression on the inactive X-chromosome in the mouse brain after Xist deletion. Epigenet. Chromatin.

[52] Carrette L.L.G., Wang C.Y., Wei C., Press W., Ma W., Kelleher R.J., Lee J.T. (2018). A mixed modality approach towards Xi reactivation for Rett syndrome and other X-linked disorders. Proc. Natl. Acad. Sci. USA.

[53] Patrat C., Ouimette J.F., Rougeulle C. (2020). X chromosome inactivation in human development. Development.

[54] Payer B., Lee J.T. (2008). X chromosome dosage compensation: How mammals keep the balance. Annu. Rev. Genet..

[55] Sahakyan A., Plath K., Rougeulle C. (2017). Regulation of X-chromosome dosage compensation in human: Mechanisms and model systems. Philos. Trans. R. Soc. Lond. B Biol. Sci..

[56] Khan S.A., Theunissen T.W. (2023). Modeling X-chromosome inactivation and reactivation during human development. Curr. Opin. Genet. Dev..

[57] Napoletano F., Baron O., Vandenabeele P., Mollereau B., Fanto M. (2019). Intersections between Regulated Cell Death and Autophagy. Trends Cell Biol..

[58] Mollereau B., Hayflick S.J., Escalante R., Mauthe M., Papandreou A., Iuso A., Celle M., Aniorte S., Issa A.R., Lasserre J.P. (2023). A burning question from the first international BPAN symposium: Is restoration of autophagy a promising therapeutic strategy for BPAN?. Autophagy.

[59] Millan-Zambrano G., Burton A., Bannister A.J., Schneider R. (2022). Histone post-translational modifications—Cause and consequence of genome function. Nat. Rev. Genet..

[60] Luger K., Dechassa M.L., Tremethick D.J. (2012). New insights into nucleosome and chromatin structure: An ordered state or a disordered affair?. Nat. Rev. Mol. Cell Biol..

[61] Lai W.K.M., Pugh B.F. (2017). Understanding nucleosome dynamics and their links to gene expression and DNA replication. Nat. Rev. Mol. Cell Biol..

[62] Turner B.M. (1993). Decoding the nucleosome. Cell.

[63] Dai Z., Ramesh V., Locasale J.W. (2020). The evolving metabolic landscape of chromatin biology and epigenetics. Nat. Rev. Genet..

[64] Wiese M., Bannister A.J. (2020). Two genomes, one cell: Mitochondrial-nuclear coordination via epigenetic pathways. Mol. Metab..

[65] McMahon R.J. (2002). Biotin in metabolism and molecular biology. Annu. Rev. Nutr..

[66] Chapman-Smith A., Cronan J.E. (1999). The enzymatic biotinylation of proteins: A post-translational modification of exceptional specificity. Trends Biochem. Sci..

[67] Leon-Del-Rio A., Leclerc D., Akerman B., Wakamatsu N., Gravel R.A. (1995). Isolation of a cDNA encoding human holocarboxylase synthetase by functional complementation of a biotin auxotroph of Escherichia coli. Proc. Natl. Acad. Sci. USA.

[68] Suzuki Y., Aoki Y., Ishida Y., Chiba Y., Iwamatsu A., Kishino T., Niikawa N., Matsubara Y., Narisawa K. (1994). Isolation and characterization of mutations in the human holocarboxylase synthetase cDNA. Nat. Genet..

[69] Wolf B., Grier R.E., Allen R.J., Goodman S.I., Kien C.L. (1983). Biotinidase deficiency: The enzymatic defect in late-onset multiple carboxylase deficiency. Clin. Chim. Acta.

[70] Ingaramo M., Beckett D. (2012). Selectivity in post-translational biotin addition to five human carboxylases. J. Biol. Chem..

[71] Choi S.W., Friso S. (2010). Epigenetics: A New Bridge between Nutrition and Health. Adv. Nutr..

[72] Hassan Y.I., Zempleni J. (2008). A novel, enigmatic histone modification: Biotinylation of histones by holocarboxylase synthetase. Nutr. Rev..

[73] Zempleni J., Chew Y.C., Bao B., Pestinger V., Wijeratne S.S. (2009). Repression of transposable elements by histone biotinylation. J. Nutr..

[74] Camporeale G., Giordano E., Rendina R., Zempleni J., Eissenberg J.C. (2006). Drosophila melanogaster holocarboxylase synthetase is a chromosomal protein required for normal histone biotinylation, gene transcription patterns, lifespan, and heat tolerance. J. Nutr..

[75] Healy S., Perez-Cadahia B., Jia D., McDonald M.K., Davie J.R., Gravel R.A. (2009). Biotin is not a natural histone modification. Biochim. Biophys. Acta.

[76] Martínez-Rubio D., Hinarejos I., Sancho P., Gorría-Redondo N., Bernadó-Fonz R., Tello C., Marco-Marín C., Martí-Carrera I., Martínez-González M.J., García-Ribes A. (2022). Mutations, Genes, and Phenotypes Related to Movement Disorders and Ataxias. Int. J. Mol. Sci..

[77] Alvarez-Cordoba M., Fernandez Khoury A., Villanueva-Paz M., Gomez-Navarro C., Villalon-Garcia I., Suarez-Rivero J.M., Povea-Cabello S., de la Mata M., Cotan D., Talaveron-Rey M. (2019). Pantothenate Rescues Iron Accumulation in Pantothenate Kinase-Associated Neurodegeneration Depending on the Type of Mutation. Mol. Neurobiol..

[78] Yue M., Charles Richard J.L., Yamada N., Ogawa A., Ogawa Y. (2014). Quick fluorescent in situ hybridization protocol for Xist RNA combined with immunofluorescence of histone modification in X-chromosome inactivation. J. Vis. Exp..

[79] Liu Y., Zhou J., Wang L., Hu X., Liu X., Liu M., Cao Z., Shangguan D., Tan W. (2016). A Cyanine Dye to Probe Mitophagy: Simultaneous Detection of Mitochondria and Autolysosomes in Live Cells. J. Am. Chem. Soc..

[80] Strober W. (2015). Trypan Blue Exclusion Test of Cell Viability. Curr. Protoc. Immunol..

[81] Tarohda T., Ishida Y., Kawai K., Yamamoto M., Amano R. (2005). Regional distributions of manganese, iron, copper, and zinc in the brains of 6-hydroxydopamine-induced parkinsonian rats. Anal. Bioanal. Chem..

[82] Georgakopoulou E.A., Tsimaratou K., Evangelou K., Fernandez Marcos P.J., Zoumpourlis V., Trougakos I.P., Kletsas D., Bartek J., Serrano M., Gorgoulis V.G. (2013). Specific lipofuscin staining as a novel biomarker to detect replicative and stress-induced senescence. A method applicable in cryo-preserved and archival tissues. Aging.

[83] Drouin-Ouellet J., Pircs K., Barker R.A., Jakobsson J., Parmar M. (2017). Direct Neuronal Reprogramming for Disease Modeling Studies Using Patient-Derived Neurons: What Have We Learned?. Front. Neurosci..

[84] Zufferey R., Nagy D., Mandel R.J., Naldini L., Trono D. (1997). Multiply attenuated lentiviral vector achieves efficient gene delivery in vivo. Nat. Biotechnol..

[85] Villanueva-Paz M., Povea-Cabello S., Villalon-Garcia I., Alvarez-Cordoba M., Suarez-Rivero J.M., Talaveron-Rey M., Jackson S., Falcon-Moya R., Rodriguez-Moreno A., Sanchez-Alcazar J.A. (2020). Parkin-mediated mitophagy and autophagy flux disruption in cellular models of MERRF syndrome. Biochim. Biophys. Acta Mol. Basis Dis..

[86] Le Boedec K. (2016). Sensitivity and specificity of normality tests and consequences on reference interval accuracy at small sample size: A computer-simulation study. Vet. Clin. Pathol..

