# Supplementary material for: Biotin Induces Inactive Chromosome X Reactivation and Corrects Physiopathological Alterations in Beta-Propeller-Protein-Associated Neurodegeneration
Source: Int J Mol Sci. 2025 Feb 4;26(3):1315. doi: 10.3390/ijms26031315 (PMC11818482; doi:10.3390/ijms26031315)
Supplement: Supplementary file 1 [file ijms-26-01315-s001.zip › ijms-3448082-supplementary.pdf]

Supplementary Figures

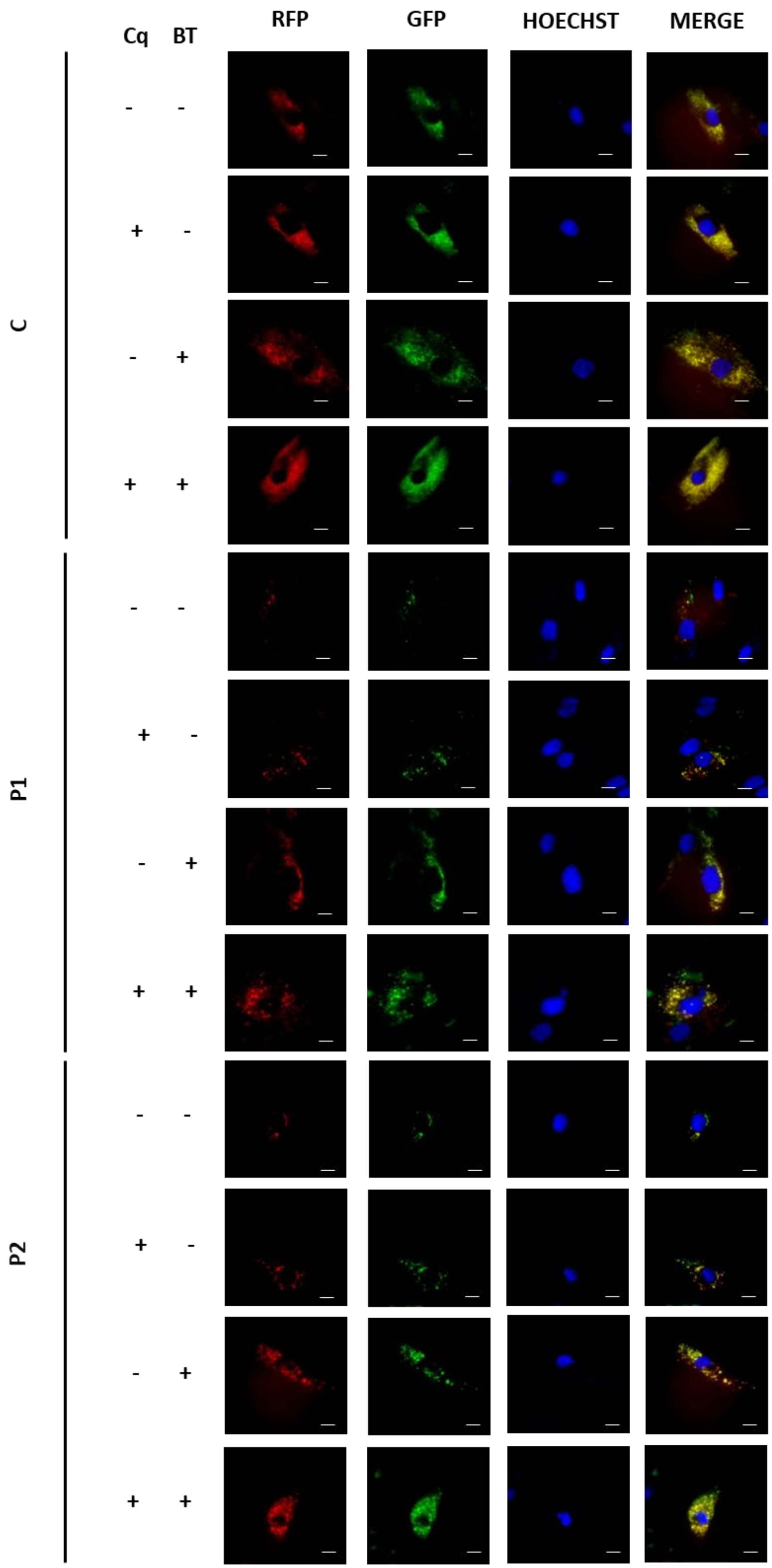
Cq
BT
RFP
GFP
HOECHST
MERGE
C
P1
P2

Figure S1. Effect of biotin supplementation on autophagosome formation in basal conditions and after autophagy inhibition by Cq. Representative fluorescence images of RFP (red), and GFP (green) of control (C) and BPAN (P1 and P2) fibroblasts in accordance with the Tandem Sensor RFP-GFP-LC3B assay in Figure 6. Scale bar: 20 µm.

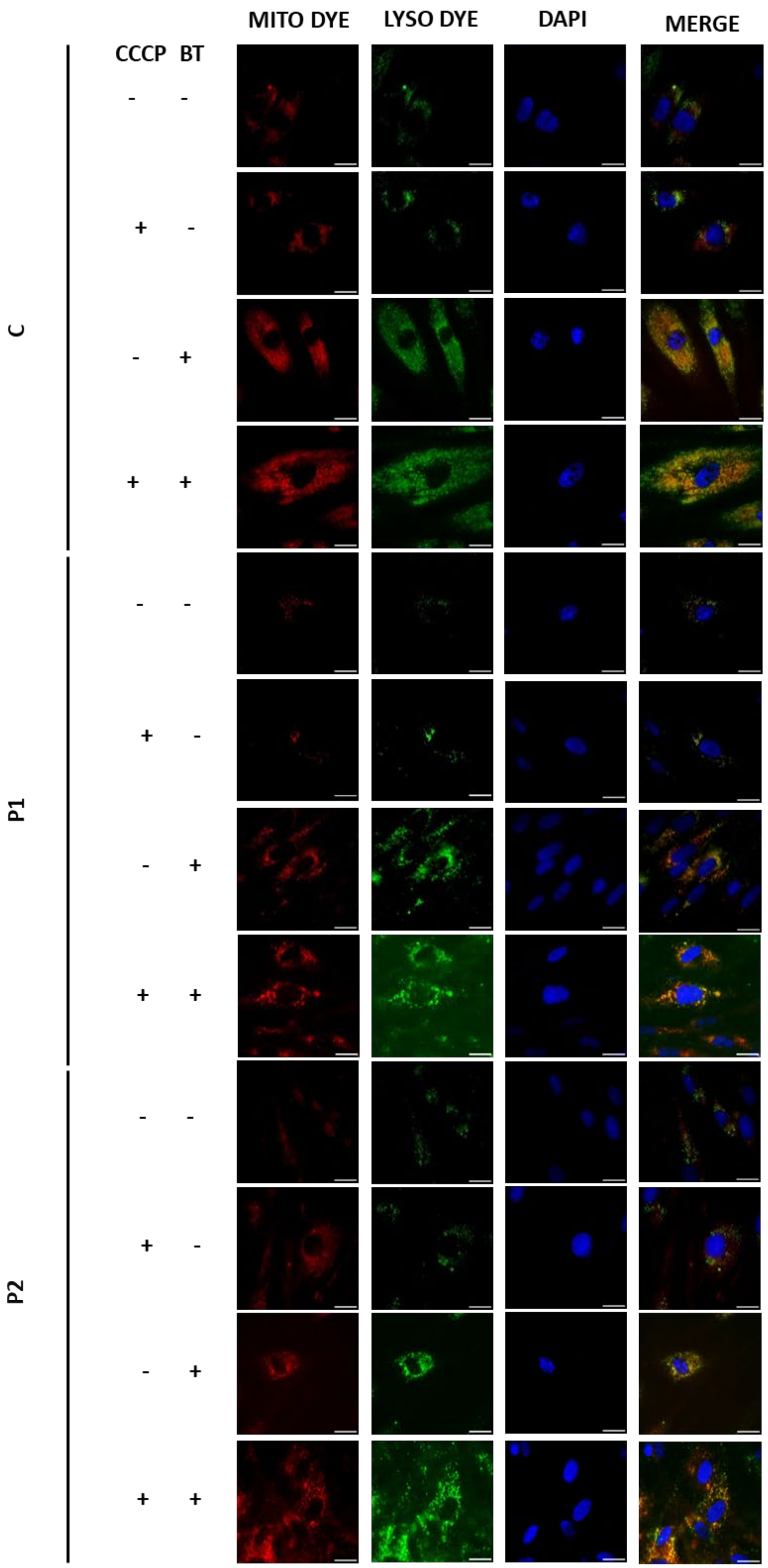

MITO DYE
LYSO DYE
DAPI
MERGE
CCCP BT
C
P1
P2

Figure S2. Effect of biotin supplementation on mitophagy activity. Representative fluorescence images of Mitophagy Dye (MITO DYE) (red) and Lyso Dye (green) of control (C) and BPAN (P1 and P2) fibroblasts in accordance with the mitophagy assay in Figure 8. Nuclei were revealed by Hoechst 33342 staining (blue). Scale bar: 20 μm.

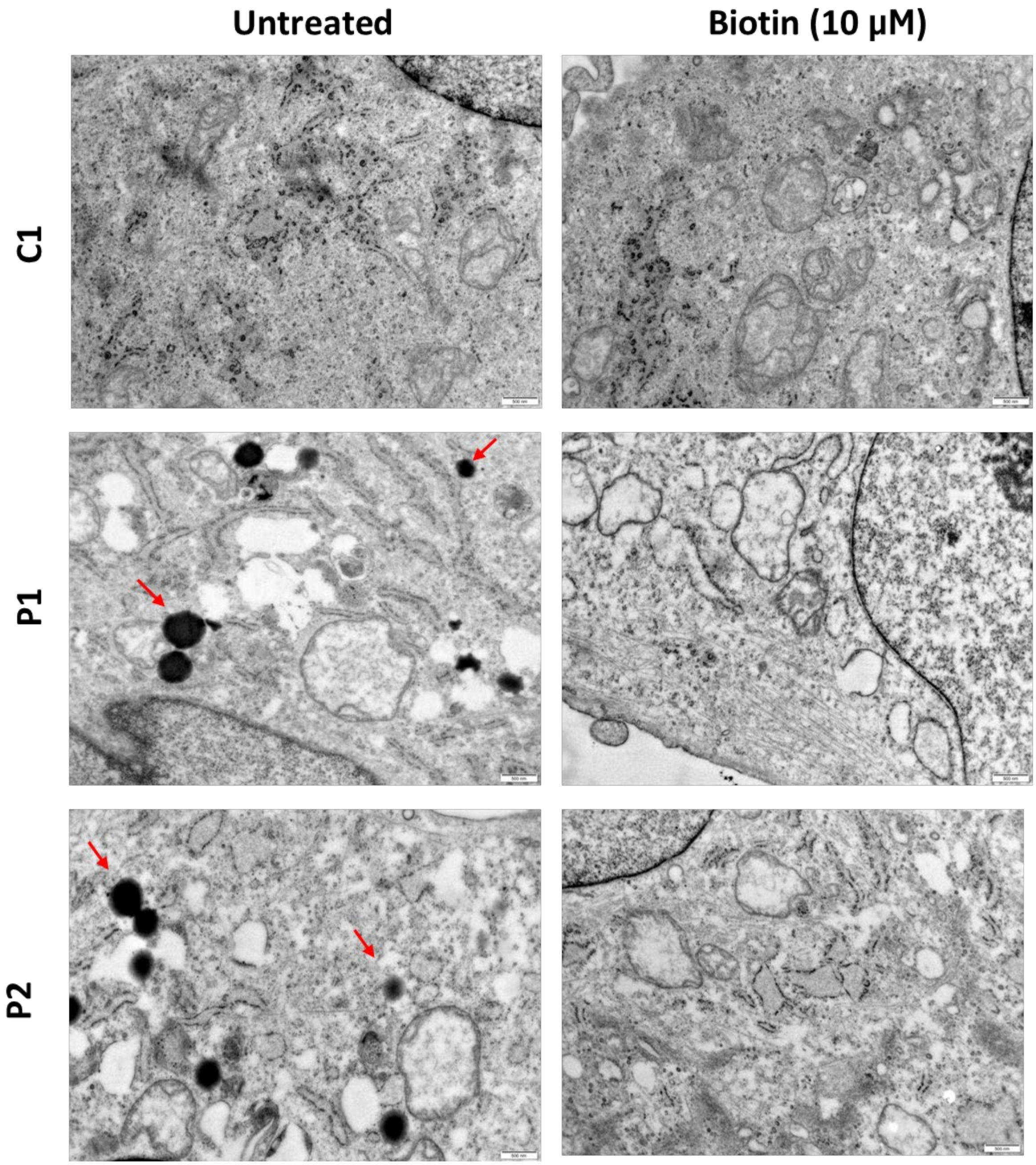

Figure S3. Effect of biotin on lipofuscin accumulation assessed by TEM. Representative electron microscopy images of lipofuscin granules (red arrows) of control (C1) and BPAN (P1 and P2) fibroblasts untreated and treated (+) with 10 μM biotin for one week in accordance with Figure 15. Scale bar: 500 nm.

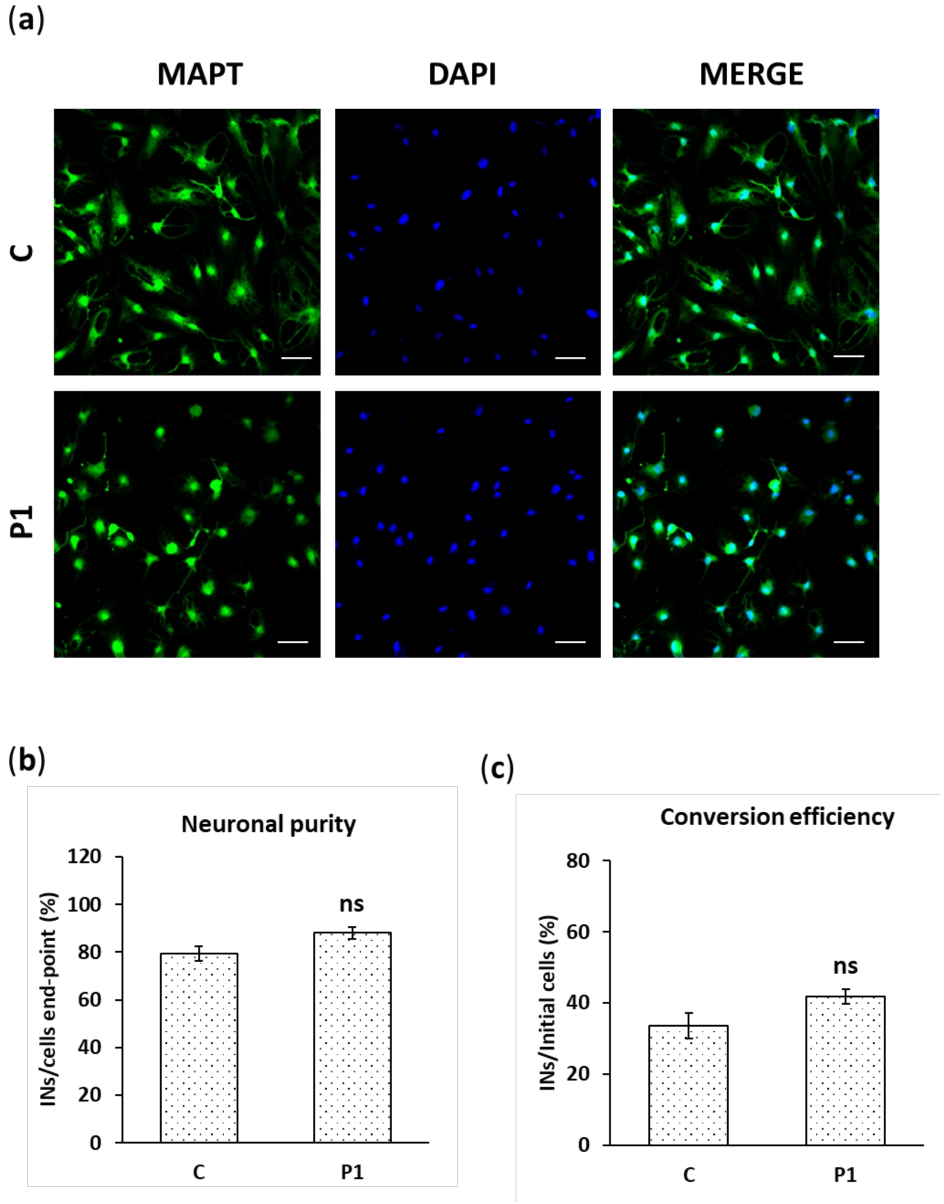

Figure S4. Neuronal purity and Conversion efficiency of iNs generated by direct reprogramming from control and BPAN fibroblasts. (**a**) Representative images of control (C) and BPAN (P1) iNs stained against MAPT (green), a neuronal marker. Nuclei were stained with 1 µg/µl DAPI. Undifferentiated cells showed only DAPI staining for the nuclei. Scale bar: 20 µm. (**b**) Neuronal purity: number of MAPT+ cells over the total of cells after reprogramming. (**c**) Conversion efficiency: number of MAPT+ cells over the total of cells seeded at the beginning of the experiment. ns: not significant.

Table S1. Sequence of fluorescently labeled oligonucleotides for *Xist* and *WDR45*.

**XIST3FW**	5′-Atto 488-TGA CAC AAG GCC AAC GAC CT-3′
**XIST3RV**	5′-Atto 488-ACA GGG GCC TTC CAT CCT TG-3′
**WDR45FW**	5′-Cy3-CAC GAT CCC AGG AGG AAC AA-3′
**WDR45RV**	5′-Cy3-GTA GAT GCG CAC ACC TGT CT-3′
